# Benchmarking Nanoscale Noncovalent Complexes at the Two-Hundred-Atom Scale with Converged Local CCSD(T)

**DOI:** 10.1021/acs.jpca.6c01097

**Published:** 2026-05-23

**Authors:** Ka Un Lao

**Affiliations:** Department of Chemistry, 6889 Virginia Commonwealth University, Richmond, Virginia 23284, United States

## Abstract

We present vL27, a benchmark data set of 27 large noncovalent complexes with sizes up to 205 atoms, designed to probe nanoscale interaction effects. Reference binding energies were computed using local coupled cluster with single, double, and perturbative triple [CCSD­(T)] extrapolated to the complete basis set (CBS) limit with VeryTightPNO thresholds and complete pair natural orbital space (CPS) extrapolation to minimize errors arising from local approximations. The MP2/CBS scheme was validated against MP2-F12, and the local CCSD­(T)/CPS protocol was benchmarked against canonical CCSD­(T), confirming the robustness of both CBS and CPS extrapolation strategies for nanoscale systems. Symmetry-adapted perturbation theory (SAPT) analysis reveals that most complexes are dispersion-dominated or exhibit mixed interaction character, even for hydrogen-bonded systems lacking π–π stacking, underscoring the central role of dispersion and many-body effects in stabilizing large assemblies. Using these benchmark data, we evaluate a broad range of electronic structure methods, semiempirical approaches, and machine learning potentials. MP2+D3-ML, B97M-D4, ωB97M-D4, and HF-3c offer the best balance of accuracy and transferability, consistently reproducing both absolute interaction energies and relative binding trends. The vL27 data set thus provides a rigorous and chemically realistic foundation for evaluating and guiding the development of computationally efficient methods for nanoscale noncovalent systems.

## Graphical abstract

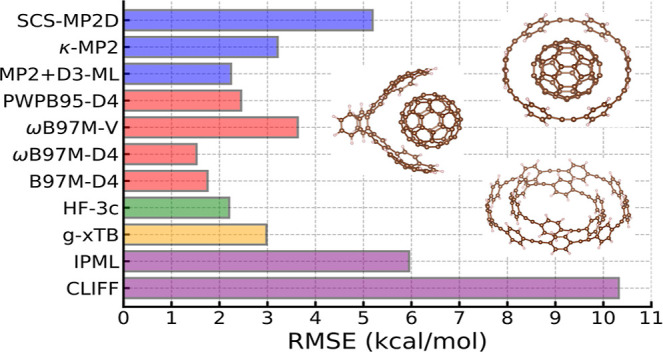

## 1 Introduction

Noncovalent interactions (NCIs) at the nanoscale play a central role in a wide range of chemical, biological, and materials processes, including molecular recognition, self-assembly, adsorption, and confinement-driven reactivity.^1–3^ As research increasingly shifts from small molecular complexes to nanoscale and mesoscale systems comprising tens to hundreds of atoms, the demand for accurate and transferable theoretical descriptions of NCIs across length scales has become increasingly urgent. However, the vast majority of widely used electronic-structure and data-driven methods have been developed, parametrized, and validated almost exclusively for small molecular systems, often under the implicit assumption that their performance can be reliably extrapolated to much larger nanoscale environments. This assumption is far from guaranteed.

At the nanoscale, NCIs are governed by physical effects that are weak, qualitatively different, or entirely absent in small systems, including many-body interactions and confinement-induced phenomena. As system size increases, these effects can fundamentally alter both the magnitude of NCIs and the balance among their underlying components, thereby breaking the fortuitous error-cancellation mechanisms that may hold for small benchmark complexes. Consequently, the accuracy of commonly used computational approaches—most notably density functional theory (DFT)—becomes increasingly unpredictable when applied to nanoscale NCIs. Indeed, several recent studies, including work from our group, have demonstrated that DFT methods performing well for small molecular systems can exhibit erratic and strongly system-dependent behavior at the nanoscale, with errors that are difficult to anticipate or systematically correct.^4–8^ These challenges are further amplified for machine learning potentials (MLPs), which are typically trained on limited chemical and size domains and may inherit—or even exacerbate—these deficiencies when deployed beyond their training regimes.

The coupled-cluster method with single, double, and perturbative triple excitations [CCSD­(T)][Bibr ref9] at the complete-basis-set (CBS) limit is widely regarded as the benchmark approach for accurately describing a broad range of chemical phenomena, including NCIs.^1,4,10–12^ Despite its steep computational cost, scaling as $O\left(N^{7}\right)$ with system size *N* and requiring substantial memory and storage resources, CCSD­(T)/CBS remains the most practical high-accuracy reference for systems beyond the reach of fully converged higher-order coupled-cluster methods. Recent studies have suggested that CCSD­(T) may slightly overestimate binding energies in certain cases,^13^ prompting renewed scrutiny of potential errors arising from truncation of the cluster operator, particularly in efforts to reconcile discrepancies between CCSD­(T) and fixed-node diffusion Monte Carlo (FN-DMC) results.^5,14,15^ Nevertheless, comparisons against higher-level coupled-cluster treatments such as CCSDT­(Q)—which are generally considered converged with respect to the cluster operator for NCIs—consistently show that CCSD­(T) yields interaction energies closest to the CCSDT­(Q) benchmark.^7^,^16–22^ As a result, CCSD­(T) remains an excellent approximation to higher-order CC theories, even for larger molecular systems.^20^ In contrast, CCSDT and CCSD­(cT) often exhibit larger deviations for systems such as π–π stacked benzene and pyridine dimers, where repulsive higher-order triple excitations and attractive connected quadruple contributions continue to yield favorable error cancellation in CCSD­(T).^7,21^ Although CCSD­(T) slightly overbinds aromatic π-stacked complexes relative to CCSDT­(Q), the magnitude of this overbinding is substantially smaller than that implied by FN-DMC results.^21,23^ For example, extrapolated calculations estimate the discrepancy between CCSD­(T) and CCSDT­(Q) for the challenging extended π-electron system C_60_[6]­CPPA to be on the order of 1 kcal/mol,^23^ whereas the reported difference between CCSD­(T) and FN-DMC binding energies for the same system is approximately 8 kcal/mol.^7,15^

Recent work has provided compelling evidence that the single-determinant (SD) fixed-node approximation in FN-DMC—by neglecting weak nondynamic correlation effects in mean-field trial wave functions—leads to systematic underbinding of π–π stacked complexes such as the benzene and coronene dimers when compared with CCSD­(T)/CBS benchmarks.^24^ This limitation underscores the need for beyond-SD nodal improvements if FN-DMC is to serve as a reliable benchmark for large noncovalent complexes.^24^ A similar conclusion applies to hydrogen-bonded systems. For instance, in the acetic acid dimer, DMC has been reported to predict stronger binding than CCSD­(T),^25,26^ indicating that fixed-node errors can manifest in opposite directions depending on the interaction type. In such cases, mitigating the fixed-node bias through beyond-SD nodal improvements requires computational costs on the order of millions of core-hours.^26^ Taken together, these findings indicate that discrepancies between CCSD­(T) and FN-DMC are unlikely to arise from truncation of the coupled-cluster operator and instead point to fixed-node errors as the dominant source.^26^ These findings therefore support CCSD­(T)/CBS as a balanced and reliable reference for NCIs, as the systematic cancellation between bonding quadruple excitations and predominantly antibonding higher-order triple contributions continues to hold relative to higher-level CCSDT­(Q) benchmarks, leading to close agreement between CCSD­(T) and CCSDT­(Q).^24^ and^26^

Against this backdrop, CCSD­(T)/CBS remains a reliable and practical reference for binding interactions, even for nanoscale noncovalent complexes where fully converged coupled-cluster treatments are computationally infeasible. Building on this premise, our group recently introduced the L14 data set,^7^ comprising 14 large noncovalent complexes (up to 113 atoms), including all seven systems from L7, three from S12L, two from S8, and two dimers relevant to the nanoaggregation of asphaltene molecules. The data set provides reference binding energies obtained using local CCSD­(T)/CBS with stringent thresholds, combined with corrections for local approximation based on canonical CCSD­(T)/def2-SVP. This protocol yields well-converged, CCSD­(T)-quality reference data at the hundred-atom scale. The L14 data set provides high-quality reference data for nanoscale NCIs, enabling the training, parametrization, and validation of lower-cost computational approaches, including DFT, semiempirical methods, force fields, and MLPs. We subsequently extended NCI benchmarking to larger and more realistic molecular assemblies—such as extended π-conjugated stacked aggregates, supramolecular complexes, and host–guest systems—resulting in the vL11 data set,^7^ consisting of 11 complexes with sizes up to 174 atoms, and the CC3@7 data set,^8^ comprising seven gas molecules binding within a nanoscale porous organic cage (up to 168 atoms), both evaluated using local CCSD­(T)/CBS with stringent thresholds.

In the present work, we further extend this effort by incorporating additional large, chemically realistic systems beyond vL11 and by systematically investigating complete pair natural orbital extrapolations^27^ under VeryTightPNO thresholds to minimize errors arising from local truncations in domain-based local pair natural orbital CCSD­(T) [DLPNO–CCSD­(T)]. In addition, the basis-set extrapolation scheme used to obtain MP2/CBS energies is validated through direct comparison with MP2-F12 calculations for large complexes. The close agreement between these two approaches confirms that conventional CBS extrapolation remains reliable and robust for large noncovalent systems. These efforts culminate in the construction of a new nanoscale benchmark data set, vL27, comprising 27 large noncovalent complexes with sizes up to 205 atoms and providing high-quality reference binding energies. Together, the L14, vL27, and CC3@7 data sets establish a rigorous foundation for understanding complex chemical environments at the nanoscale and serve as indispensable resources for the development, assessment, refinement, training, parametrization, and validation of computationally efficient methods in this regime. Moreover, as MLPs become increasingly prevalent in computational chemistry, these nanoscale benchmark data sets offer high-quality training and validation data that are essential for achieving chemical accuracy in large, chemically realistic systems.

## 2 Computational Methods

Given the high computational cost of CCSD­(T), focal-point approaches are commonly employed to obtain CCSD­(T)/CBS-quality energies in a computationally efficient manner

1 $$E^{CCSD\left(T\right)/CBS}=E^{HF/CBS}+E_{corr}^{MP2/CBS}+\Delta E_{corr}^{CCSD\left(T\right)}$$

where the HF/CBS and MP2/CBS terms denote the Hartree–Fock energy and MP2 correlation energy at the CBS limit, respectively. Previous studies have demonstrated that the HF/CBS limit can be reliably reached using aug-cc-pVQZ (aQZ)^28,29^ or heavy-aug-cc-pVQZ (haQZ)^30^ basis sets, even for large noncovalent complexes.^5^ Accordingly, aQZ or haQZ basis sets are employed in this work to obtain well-converged Hartree–Fock energies. The MP2 correlation energy at the CBS limit is extrapolated using the robust inverse cubic formula,^31^ based on aug-cc-pVTZ/aug-cc-pVQZ [a­(T,Q)­Z] or heavy-aug-cc-pVTZ/heavy-aug-cc-pVQZ [ha­(T,Q)­Z] basis-set pairs.

Our group previously carried out the first systematic assessment of CBS extrapolation parameters for MP2 correlation energies in large noncovalent complexes using the L7 benchmark set, with MP2/a­(T,Q)­Z extrapolation employing the inverse cubic scheme taken as the reference. It should be noted that the commonly adopted extrapolation parameters for smaller basis sets were originally optimized against reference energies of small molecules containing at most two heavy atoms.^32^ We showed that when MP2/a­(T,Q)­Z extrapolation is not feasible, only the ha­(T,Q)­Z extrapolation using the same inverse cubic formula yields well-converged MP2 correlation energies, with residual errors on the order of 0.01 kcal/mol.^5^ In this work, MP2 energies obtained using the a­(T,Q)­Z extrapolation scheme are benchmarked against MP2-F12 results with triple-ζ and quadruple-ζ basis sets for large noncovalent complexes in the L7 benchmark set. The MP2-F12 calculations provide highly converged reference values,^33^ enabling a critical assessment of whether the a­(T,Q)­Z extrapolation scheme remains reliable for large noncovalent systems.

The ΔCCSD­(T) correlation correction represents the difference between CCSD­(T) and MP2 correlation energies and is typically evaluated using triple-ζ basis sets, which are generally sufficient to achieve convergence of ΔCCSD­(T).^34,35^ However, canonical CCSD­(T) calculations with triple-ζ basis sets remain prohibitively expensive for systems approaching the hundred-atom scale considered in this work. To alleviate this computational bottleneck, we previously introduced a partitioning of the ΔCCSD­(T) correlation correction into two contributions^7^

2 $$\Delta E_{corr}^{CCSD\left(T\right)}=\Delta E_{corr}^{CCSD\left(T\right)/small}+\Delta \Delta E_{corr}^{CCSD\left(T\right)}$$

The first term, Δ*E*_corr_^CCSD(T)/small^, corresponds to the difference between CCSD­(T) and MP2 correlation energies evaluated with a double-ζ def2-SVP basis set

3 $$\Delta E_{corr}^{CCSD\left(T\right)/small}=E_{corr}^{CCSD\left(T\right)/\mathrm{def}2‐SVP}-E_{corr}^{MP2/\mathrm{def}2‐SVP}$$

Canonical CCSD­(T)/def2-SVP calculations are ideal for evaluating this term, as employed for the reference calculations of our previous L14 data set.^7^ However, DLPNO–CCSD­(T_1_) with VeryTightPNO thresholds, as used in our previous vL11 data set,^7^ and DLPNO–CCSD­(T_1_) with VeryTightPNO thresholds combined with CPS extrapolation, as employed in the present vL27 data set, are also sufficiently accurate due to their close agreement with canonical CCSD­(T) results, even for the relatively large systems considered in this work. The second term, ΔΔ*E*_corr_^CCSD(T)^, accounts for the basis-set correction associated with extending the post-MP2 correlation contribution from the double-ζ to the triplet-ζ level. This correction is evaluated using DLPNO–CCSD­(T) to mitigate the computational cost of triple-ζ calculations and is given by

4 $$\Delta \Delta E_{corr}^{CCSD\left(T\right)}=\left(E_{corr}^{DLPNO‐CCSD\left(T\right)/\mathrm{def}2‐TZVPP}-E_{corr}^{MP2/\mathrm{def}2‐TZVPP}\right)-\left(E_{corr}^{DLPNO‐CCSD\left(T\right)/\mathrm{def}2‐SVP}-E_{corr}^{MP2/\mathrm{def}2‐SVP}\right)$$

It is well established that binding energies obtained from local CCSD­(T) methods are sensitive to the threshold parameters controlling the accuracy of the local approximation.^5,6,15^ For example, tightening the DLPNO thresholds from TightPNO (TCutPairs = 10^–5^, TCutPNO = 10^–7^, TCutMKN = 10^–3^) to VeryTightPNO (TCutPairs = 10^–6^, TCutPNO = 10^–8^, TCutMKN = 10^–4^) at the DLPNO–CCSD­(T_1_)/def2-TZVPP level for the coronene dimer in the L7 benchmark set—corresponding to one of the terms in eq 4—leads to changes in binding correlation energies exceeding 2 kcal/mol, well beyond the chemical accuracy target of 1 kcal/mol.^7^ In contrast, this sensitivity is largely suppressed in the basis-set correction term ΔΔ*E*_corr_^CCSD(T)^ in eq 4. For instance, ΔΔ*E*_corr_^CCSD(T)^ changes by only 0.04 kcal/mol for the C2C2PD complex when tightening the DLPNO thresholds from TightPNO to VeryTightPNO.^7^ This reduced sensitivity is attributed to cancellation of local-approximation errors between DLPNO–CCSD­(T_1_) calculations performed with def2-TZVPP and def2-SVP basis sets in eq 4, indicating that large local errors are effectively mitigated in ΔΔ*E*_corr_^CCSD(T)^. As a result, the scheme defined in eq 2 enables a substantial reduction in the computational cost of canonical CCSD­(T) calculations by restricting them to a double-ζ basis set (def2-SVP), while incorporating post-MP2 basis-set corrections up to the triple-ζ level through DLPNO–CCSD­(T_1_), without requiring stringent convergence of the local approximation. We further examined the effect of increasing the basis-set size in both Δ*E*_corr_^CCSD(T)/small^ and ΔΔ*E*_corr_^CCSD(T)^, observing only marginal changes in the resulting correlation energies.^7^ These results confirm the robustness of the proposed protocol—employing def2-SVP for Δ*E*_corr_^CCSD(T)/small^ and def2-TZVPP/def2-SVP for ΔΔ*E*_corr_^CCSD(T)^—in reliably approximating Δ*E*_corr_^CCSD(T)^ at the canonical CCSD­(T) level with triple-ζ basis sets. This scheme was previously employed to construct reference binding energies for the nanoscale L14 benchmark set.^7^

Despite the use of the def2-SVP double-ζ basis set, canonical CCSD­(T) calculations remain computationally prohibitive for the even larger nanoscale systems considered in this work. Consequently, local CCSD­(T) approaches are required in eq 3 for all 27 large complexes considered in this work, although careful control of the threshold parameters governing the local approximation remains essential. Relative to canonical CCSD­(T)/def2-SVP reference energies for the 14 complexes in the L14 benchmark set,^7^ DLPNO–CCSD­(T_1_) with the TightPNO threshold systematically overestimates binding energies for all complexes except PHE, yielding a mean absolute error (MAE) of 1.05 kcal/mol and a maximum absolute error (MAX) of 2.44 kcal/mol, as shown in Table 1. The largest deviation is observed for the stacking of the guanine–cytosine pair on circumcoronene (C3GC). Tightening the thresholds to VeryTightPNO substantially improves the agreement, reducing the MAE to 0.39 kcal/mol and the MAX to 0.65 kcal/mol, with the coronene dimer (C2C2PD) exhibiting the largest residual error. Notably, VeryTightPNO results systematically underestimate binding energies across the data set. These results reinforce earlier findings that PNO thresholds tighter than TightPNO are required to achieve chemical accuracy for large supramolecular complexes.^36^ Furthermore, we have demonstrated that DLPNO–CCSD­(T_1_) with VeryTightPNO thresholds outperforms other DLPNO schemes commonly employed in the literature as reference methods,^37–40^ delivering benchmark-quality interaction energies even for large complexes.^7^

**Table 1:** Mean Absolute Errors (MAE) and Maximum Absolute Errors (MAX) in kcal/mol, for DLPNO–CCSD­(T_1_)/def2-SVP Using the TightPNO (TCutPairs = 10^–5^, TCutPNO = 10^–7^, TCutMKN = 10^–3^) and VeryTightPNO (TCutPairs = 10^–6^, TCutPNO = 10^–8^, TCutMKN = 10^–4^) Thresholds, as well as the 6/7 CPS Extrapolation Based on TightPNO and 7/8 CPS Extrapolation Based on VeryTightPNO, Relative to Canonical CCSD­(T)/def2-SVP Reference Values for the 14 Complexes in the L14 Benchmark Set

method	threshold	CPS	MAE	MAX
DLPNO–CCSD(T_1_)	TightPNO	–	1.05	2.44
DLPNO–CCSD(T_1_)	VeryTightPNO	–	0.39	0.65
DLPNO–CCSD(T_1_)	TightPNO	6/7	1.58	3.23
DLPNO–CCSD(T_1_)	VeryTightPNO	7/8	0.11	0.37

To further improve the accuracy of local CCSD­(T) calculations, an extrapolation scheme has been proposed to approximate the complete PNO (CPS) limit for DLPNO methods^27^

5 $$E_{corr}^{CPS}=E_{corr}^{X}+1.5\times \left(E_{corr}^{Y}-E_{corr}^{X}\right)$$

where *E*_corr_^CPS^ denotes the DLPNO–CCSD­(T_1_) correlation energy at the CPS limit. The quantities *E*_corr_^*X*^ and *E*_corr_^*Y*^ correspond to DLPNO–CCSD­(T_1_) correlation energies computed using TCutPNO thresholds of 10^–*X*^ and 10^–*Y*^, respectively, with *Y* = *X* + 1. All other DLPNO computational settings are kept identical, such that the two calculations differ only in the TCutPNO threshold. This CPS extrapolation scheme has been shown to improve upon TightPNO results for NCIs,^41^ reaction energies, barrier heights,^42–44^ and spin-state splittings.^45^ However, because the scheme relies on an empirically determined parameter optimized for small systems, its applicability to large supramolecular complexes remains uncertain.

Here, we first applied the CPS extrapolation scheme using TCutPNO values of 10^–6^ and 10^–7^ while retaining all other DLPNO settings at the TightPNO level for the 14 complexes in the L14 benchmark set. This 6/7 extrapolation significantly degrades the performance, yielding an MAE of 1.58 kcal/mol and an MAX of 3.23 kcal/mol, with the largest error again observed for C3GC, as indicated in Table 1. In this case, the CPS-extrapolated energies systematically overestimate binding strengths and perform worse than TightPNO results without CPS extrapolation. In contrast, applying the CPS extrapolation scheme using TCutPNO values of 10^–7^ and 10^–8^ with all other DLPNO settings at the VeryTightPNO level leads to a substantial improvement in accuracy. This 7/8 extrapolation yields an MAE of 0.11 kcal/mol and a maximum deviation of 0.37 kcal/mol, with the largest residual error observed for C2C2PD. Importantly, the 7/8 CPS extrapolation consistently improves upon the VeryTightPNO results for all 14 complexes. These results demonstrate that the two-point CPS extrapolation scheme remains effective for large noncovalent complexes when sufficiently tight local thresholds are employed, as in the 7/8 extrapolation based on the VeryTightPNO settings used here. This provides a cost-effective strategy for further reducing local-approximation errors in DLPNO-based calculations. Consequently, our findings indicate that very tight local thresholds are not only necessary to achieve chemical accuracy for NCIs, but also provide the foundation required to obtain reliably converged CPS-extrapolated results.

Although similar DLPNO error trends have been observed across small and large basis sets in DLPNO-MP2 studies,^36^ their transferability to DLPNO–CCSD­(T) is not guaranteed. Accordingly, the present DLPNO–CCSD­(T) analysis is based solely on the def2-SVP double-ζ basis set, and the observed performance may not fully extend to larger basis sets, such as triple-ζ. A definitive assessment of the transferability of DLPNO errors from small to large basis sets would require canonical CCSD­(T) calculations with triple-ζ basis sets; however, such calculations are computationally prohibitive for the large noncovalent complexes considered here.

Table 1 summarizes the MAE and MAX for DLPNO–CCSD­(T_1_) using TightPNO and VeryTightPNO thresholds, as well as the 6/7 CPS (TightPNO) and 7/8 CPS (VeryTightPNO) extrapolation schemes, relative to canonical CCSD­(T)/def2-SVP reference energies for the L14 benchmark set. The individual CCSD­(T) binding energies for all complexes at the different settings are provided in Table S1. Because DLPNO–CCSD­(T_1_) with VeryTightPNO and the 7/8 CPS extrapolation achieves sub kcal/mol accuracy relative to canonical CCSD­(T) for all complexes in the L14 benchmark set, it is adopted in place of canonical CCSD­(T) in the evaluation of Δ*E*_corr_^CCSD(T)/small^ [eq 3] for all 27 large noncovalent complexes comprising the vL27 data set introduced below.

A nanoscale binding data set consisting of 27 very large noncovalent complexes, with system sizes of up to 205 atoms, has been assembled and is denoted as vL27. All binding energies in the vL27 data set are computed at the DLPNO–CCSD­(T_1_)/CBS level, employing the VeryTightPNO thresholds in combination with the 7/8 CPS extrapolation scheme, based on eqs 1–5. The vL27 data set includes all 11 complexes from the previously reported vL11 set, comprising a pincer complex with 2,4,7-trinitro-9-fluorenone (S12L-3a); a buckycatcher complex with C_60_ (S12L-4a); two amine macroycyle complexes with benzoquinone and glycine anhydride (S12L-5a and S12L-5b); the U-DPy dimer (CiM-a); the poly­(benzyl ether) dendrimer dimer (CiM-c); a cyclic peptide dimer (CiM-d); the triglutamyl-1,3,5-benzenetricarboxamide dimer (CiM-e); the DNA-ellipticine intercalation complex (DNA-ellipticine), which is the only charged system in this study, featuring –2 charges on the two phosphate groups; the gas-phase C_60_ dimer at the equilibrium interfullerene distance (C_60_–C_60_), and a C_60_ buckyball encapsulated inside a [6]-cycloparaphenyleneacetylene ring (C_60_@[6]­CPPA). In addition, 16 new complexes from previous studies are incorporated into vL27 in this work. They include the cucurbit[7]­uril host–guest complex with 1-hydroxyadamantane (S12L-7b) from the S12L data set;^46^ nine complexes from the S30L data set,^47^ namely two molecular tweezer complexes with 2,4,7-trinitro-9-fluorenone and tetracyanoquinodimethane as guests (S30L-5 and S30L-6); two ring-in-ring complexes involving [5]­cycloparaphenyleneacetylene (5CPPA) in 8CPPA (S30L-7) and 6CPPA in 9CPPA (S30L-8); two resorcin[4]­arene-based container complexes with morpholine and tioxane as guests (S30L-13 and S30L-14); two β-cyclodextrin complexes with cyclopentanol and cyclooctanol as guests (S30L-19 and S30L-20), and one quadruple hydrogen-bond complex featuring a DAAD-ADDA array in which both host and guest act as double donors and double acceptors (S30L-22). These additional structures were selected from the S12L and S30L data sets based on available computational resources, given the time- and memory-intensive nature of calculations for large noncovalent complexes. The data set further includes six C_60_ complexes with polycyclic aromatic hydrocarbons—coronene (Cor), corannulene (Coran), sumanene (Suman), triazasumanene (3NSuman), pentachlorocorannulene (PClCoran), and decachlorocorannulene (DClCoran)^48^—to enable a systematic investigation of the binding order of C_60_ with different host complexes. These systems, together with additional cases from S12L and S30L involving the same host complex interacting with different guests, were deliberately included to facilitate a meaningful assessment of relative binding trends. It is well-known that some methods can yield accurate absolute binding energies while failing to reproduce correct relative rankings.^8^

All 27 complexes in the vL27 data set, together with their dominant binding character as determined from symmetry-adapted perturbation theory (SAPT) analysis, are illustrated in Figure 1. For each complex, the most accurate level of theory feasible within available computational resources was selected for the focal-point approach defined in eq 1, as summarized in Table 2. Specifically, aQZ or haQZ basis sets were employed for the Hartree–Fock component, a­(T,Q)­Z or ha­(T,Q)­Z for the MP2 correlation energy, DLPNO–CCSD­(T_1_)/def2-SVP with VeryTightPNO and the 7/8 CPS extrapolation for Δ*E*_corr_^CCSD(T)/small^, and DLPNO–CCSD­(T_1_)/def2-TZVPP with VeryTightPNO or TightPNO to account for basis-set effects in the post-MP2 correlation contribution.

**Figure 1:**
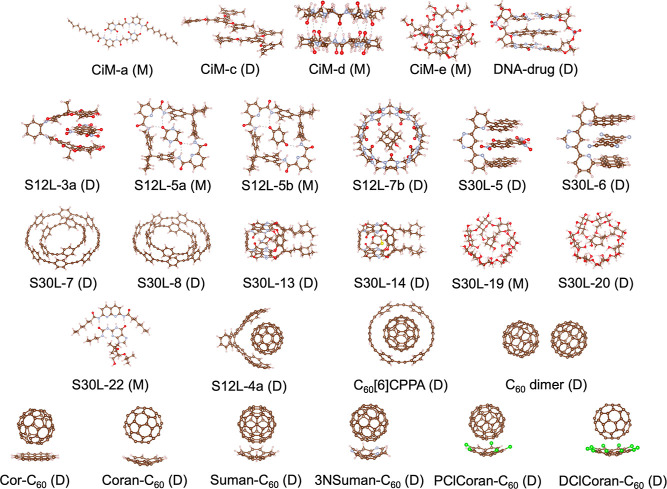
The vL27 data set comprises 27 large noncovalent complexes with system sizes of up to 205 atoms. The data set includes five complexes from S12L (3a, 4a, 5a, 5b, and 7b), nine complexes from S30L (5, 6, 7, 8, 13, 14, 19, 20, and 22), four complexes from CiM13 (CiM-a, CiM-c, CiM-d, and CiM-e), the DNA-ellipticine intercalation complex, the C_60_ dimer (C_60_–C_60_), C_60_ encapsulated in a [6]­cycloparaphenyleneacetylene ring (C_60_@[6]­CPPA), and six C_60_-PAH complexes involving coronene (Cor), corannulene (Coran), sumanene (Suman), triazasumanene (3NSuman), pentachlorocorannulene (PClCoran), and decachlorocorannulene (DClCoran). Each system is annotated by its dominant binding character based on SAPT­(KS)+MBDrev-ML/def2-TZVPPD analysis: **D** denotes dispersion-dominated complexes (|*E*_disp_/*E*_elst_| > 2), **E** denotes electrostatics-dominated complexes (|*E*_disp_/*E*_elst_| < 0.5), and **M** denotes mixed binding character, where both contributions are significant.

**Table 2:** Computational Protocols Used to Evaluate Noncovalent Binding Energies for the 27 Complexes in the vL27 Data Set at the DLPNO–CCSD­(T_1_)/CBS Level, Employing the VeryTightPNO Thresholds and the 7/8 CPS Extrapolation Scheme^c^

vL27 complexe	HF/CBS	MP2/CBS	Δ*E*_corr_^CCSD(T)/small^ ^a^	ΔΔ*E*_corr_^CCSD(T)^ ^b^
CiM-a	haQZ	ha(T,Q)Z	DLPNO–CCSD(T_1_) (VeryTightPNO) 7/8 CPS extrapolation	DLPNO–CCSD(T_1_) (VeryTightPNO)
CiM-c, CiM-d, CiM-e S12L-4a, DNA-ellipticine C_60_@[6]CPPA, C_60_–C_60_ S12L-7b, S30L-5, S30L-6, S30L-7, S30L-8, S30L-13, S30L-14, S30L-19, S30L-20, S30L-22, Cor–C_60_, Coran–C_60_, Suman–C_60_, 3NSuman–C_60_, PClCoran–C_60_, DClCoran–C_60_	haQZ	ha(T,Q)Z	DLPNO–CCSD(T_1_) (VeryTightPNO) 7/8 CPS extrapolation	DLPNO–CCSD(T_1_) (TightPNO)
S12L-3a, S12L-5a, S12L-5b	aQZ	a(T,Q)Z	DLPNO–CCSD(T_1_) (VeryTightPNO) 7/8 CPS extrapolation	DLPNO–CCSD(T_1_) (TightPNO)

a def2-SVP.

b def2-TZVPP/def2-SVP.

c The table summarizes the basis sets used for the Hartree–Fock component, the extrapolation schemes applied to obtain converged MP2 correlation energies, the basis set and DLPNO–CCSD­(T_1_) level used to compute Δ*E*_corr_^CCSD(T)/small^ in eq 3, and the large and small basis sets, along with the DLPNO thresholds, used to evaluate ΔΔ*E*_corr_^CCSD(T)^ in eq 4.

The newly proposed vL27 data set is used to assess the performance of a range of widely employed computational methods for describing NCIs in nanoscale complexes. The methods considered include MP2 and several of its improved variants, such as SCS-MP2,^49^ SCS­(MI)-MP2,^50^ MP2D,^51^ SCS-MP2D,^52^ and the regularized κ-MP2 method with κ = 1.1, as recommended in ref 53. In addition, our recently developed data-driven MP2+*ai*D­(CCD)—also referred to as MP2+D3-ML—is included in this evaluation.^54,55^ With the exception of MP2+D3-ML and κ-MP2, all MP2-based binding energies are extrapolated to the CBS limit. For MP2+D3-ML and κ-MP2, accurate results are obtained using only the aTZ basis set, substantially reducing the computational cost. The MP2+D3-ML approach is distinguished by its exceptional accuracy in describing NCIs, consistently outperforming other MP2-based methods even for nanoscale systems, while requiring only aTZ-quality calculations.^55^ Conceptually, this method is analogous to SAPT2+(CCD)­δMP2 with the inclusion of the *E*_disp_^(30)^ term, or to SAPT2+(3)­(CCD)­δMP2 without the *E*_elst_^(13)^ contribution. However, in MP2+D3-ML, the explicit evaluation of higher-order SAPT dispersion terms is replaced by a data-driven model that efficiently and accurately captures dispersion interactions, thereby avoiding the computationally prohibitive $O\left(N^{7}\right)$ scaling associated with such calculations.^55^

In addition, the favorable error cancellation of SAPT0 with the jun-cc-pVDZ (jaDZ) basis set^56^ is assessed. A broad range of DFT methods is also evaluated, including contemporary functionals such as ωB97M-V,^57^ ωB97X-V,^58^ B97M-V,^59^ and B97M-rV^60^ as well as their corresponding D4 variants: ωB97X-D4,^61^ ωB97M-D4,^61^ ωB97X-D4rev,^62^ ωB97M-D4rev,^63^ and B97M-D4.^61^ We further consider a selection of dispersion-corrected functionals, including B3LYP+D4,^64–66^ PBE0+D4,^66,67^ PBE0+MBD,^67,68^ PBE+D4,^66,69^ ωB97X-D,^70^ and ωB97X-D3.^71^ Minnesota functionals—MN15,^72^ M06–2X-D3,^73,74^ M06-L-D4,^66,75^ and PW6B95-D4 ^66,76^ —as well as double-hybrid functionals, including PWPB95-D4,^66,77^ revDSD-PBEP86-D4,^78^ and ωB97M(2),^79^ are also examined. The performance of efficient composite electronic-structure methods—HF-3c,^80^ PBEh-3c,^81^ B97–3c,^82^ r^2^SCAN-3c,^83^ and ωB97X-3c^62^—as implemented in ORCA, is additionally investigated for nanoscale complexes. Semiempirical approaches, including PM6-D3H4,^84^ DFTB3-D3H5,^85^ GFN2-xTB,^86^ and g-xTB^87^ are employed to compute binding energies for nanoscale systems in the vL27 data set. The hybrid PM6-ML method,^88^ which combines PM6 with an MLP, is further examined to assess its transferability to nanoscale NCIs. Finally, based on our previous findings that the ANI family of MLPs underperforms for predicting binding energies in nanoscale systems,^55^ only the IPML^89^ and CLIFF^90^ MLPs are considered in this work, using their respective standalone implementations.

SAPT­(KS), in which symmetry-adapted perturbation theory is formulated using Kohn–Sham molecular orbitals derived from the long-range-corrected LRC-ωPBE density functional^91^ in conjunction with the def2-TZVPPD basis set, is employed as a computationally efficient approach for incorporating intramolecular electron correlation. Combined with our atomic-orbital-based SAPT implementation,^92^ SAPT­(KS) is used to compute the nondispersion interaction energy components—namely electrostatics, exchange, and induction—for all complexes in the vL27 data set. The system-specific, global-density-dependent^92,93^ range–separation parameter for LRC-ωPBE is determined independently for each monomer within a dimer using our accurate and efficient XGBoost-based ML model,^94^ available through our GitHub repository.^95^ This procedure enforces the correct asymptotic behavior of the exchange–correlation functional for each monomer,^96^ thereby enhancing the reliability of the resulting SAPT­(KS) interaction components. To account for dispersion interactions, a series of *ab initio* dispersion potentials (*ai*D) have been developed to reproduce dispersion energies obtained from higher-level SAPT calculations.^54,97–101^ The resulting atomic-orbital-based SAPT­(KS)+*ai*D framework exhibits only cubic scaling with respect to monomer size.^92^ In this work, several SAPT­(KS)+*ai*D variants are evaluated for all complexes in the vL27 data set, including SAPT­(KS)+*ai*D3,^98^ SAPT­(KS)+MBD,^99,100^ our recently refitted MBD model SAPT­(KS)+MBDrev,^54^ as well as two ML-corrected dispersion potentials, SAPT­(KS)+D3-ML and SAPT­(KS)+MBDrev-ML.^54^ A δHF correction term is included to account for higher-order polarization effects beyond second order.

All MP2 and SAPT0 calculations were performed using PSI4,^102^ while all SAPT­(KS)+*ai*D calculations were carried out using a development version of Q-Chem.^103^ The ML-corrected dispersion potentials, D3-ML and MBDrev-ML, were obtained from the freely available GitHub repository.^104^ DLPNO–CCSD­(T_1_), MP2-F12, and MP2-F12D calculations were carried out with ORCA.^105^ For MP2-F12 and MP2-F12D, the cc-pV*n*Z-F12 basis sets (*n* = D, T, Q) were employed together with the corresponding cc-pV*n*Z-F12-CABS auxiliary basis sets. The resolution-of-the-identity (RI) or density-fitting approximation was applied in both the self-consistent field and post-Hartree–Fock calculations using the appropriate auxiliary basis sets. For MP2-F12 and MP2-F12D, larger auxiliary basis sets—specifically, cc-pV*n*Z (*n* = T, Q, 5)—were used for RI-JK in the Hartree–Fock step and for RI in the evaluation of correlation energies. All DFT calculations employing the def2-TZVPPD basis set were carried out with Q-Chem,^103^ except for B3LYP+D4, PBE0+D4, PBE+D4, which were performed in PSI4, and PW6B95-D4, PWPB95-D4, and revDSD-PBEP86-D4, which were evaluated using ORCA. Additionally, binding energies for the three double-hybrid functionals PWPB95-D4, revDSD-PBEP86-D4, and ωB97M(2), were extrapolated to the CBS limit using an inverse-cubic extrapolation scheme based on the def2-TZVPP and def2-QZVPP basis sets.

Semiempirical calculations with PM6-D3H4, PM6-ML, and DFTB3-D3H5 were performed using the Cuby4 framework,^106^ interfaced with MOPAC^107^ and DFTB+,^108^ respectively. GFN2-xTB calculations were carried out with ORCA interfaced to the xtb program package,^109^ while g-xTB calculations were performed using its standalone implementation.^87^ All DFT and post-Hartree–Fock calculations were counterpoise (CP) corrected to remove basis-set superposition error (BSSE) except for MP2-F12 and MP2-F12D, for which both uncorrected and CP-corrected binding energies are reported. Core electrons were kept frozen in all correlation calculations. Three-body Axilrod–Teller–Muto (ATM) contributions were included in the D4 dispersion correction using the Becke–Johnson damping function. The ATM term is included in the D3 correction for M06–2X but not for ωB97X-D3, consistent with the original parametrization of the latter functional. All binding energies were computed using monomer geometries extracted from the optimized complexes, without further relaxation of the monomer structures.

It should be noted that the most time- and memory-intensive calculations for the large complexes considered in this work arise from DLPNO–CCSD­(T_1_)/def2-TZVPP with TightPNO or DLPNO–CCSD­(T_1_)/def2-SVP with VeryTightPNO. For example, one of the most demanding cases, the DClCoran–C_60_ dimer, requires approximately 3 weeks on 8 cores (AMD EPYC 9474F, 3.60 GHz), with about 3 TB of memory and 11.5 TB of SSD storage at the DLPNO–CCSD­(T_1_)/def2-TZVPP level with TightPNO using ORCA. Consequently, evaluating all binding energy components in eq 1 for a single large noncovalent complex on a high-memory, high-storage node may require several months to obtain a well-converged CCSD­(T)/CBS result.

## 3 Results and Discussion

### 3.1 MP2/CBS versus MP2-F12 and MP2-F12D for the L7 Data Set

A physically more appealing alternative to conventional basis-set extrapolation is the use of explicitly correlated methods, which are well-known to deliver near-CBS limit correlation energies when combined with substantially smaller orbital basis sets. To assess the suitability of CBS extrapolation schemes for large noncovalent complexes, we employed MP2-F12 and its computationally efficient variant MP2-F12D in conjunction with the cc-pV*n*Z-F12 basis sets (*n* = D, T, Q), hereafter denoted as DZ, TZ, and QZ, respectively, to approximate MP2 energies at the CBS limit for the seven complexes in the L7 data set. These results are compared with MP2/CBS reference energies obtained using the robust inverse-cubic extrapolation formula based on the a­(T,Q)­Z scheme, as summarized in Tables S2 and S3.

MP2-F12D shows reasonable agreement with MP2-F12 at the DZ level, with an MAE of 0.10 kcal/mol and a MAX of 0.22 kcal/mol, which are further reduced to an MAE of 0.04 kcal/mol and a MAX of 0.21 kcal/mol upon application of CP correction. Beginning at the TZ level, MP2-F12D yields essentially identical results to MP2-F12, with deviations not exceeding 0.02 and 0.01 kcal/mol for the TZ and QZ basis sets, respectively. With CP correction applied, MP2-F12D and MP2-F12 produce indistinguishable binding energies at both the TZ and QZ levels. In the following discussion, only MP2-F12D results are therefore considered and compared with MP2/CBS values, as MP2-F12/QZ calculations are computationally prohibitive for the C3GC complex.

For MP2-F12D calculations without CP correction, increasing the basis set size from DZ to TZ leads to systematically weaker binding for all seven L7 complexes, with an average change of 0.44 kcal/mol and a maximum change of 1.06 kcal/mol observed for C3GC. In contrast, when CP correction is applied, the binding energies increase upon going from DZ to TZ, with the same average change of 0.44 kcal/mol and a reduced maximum change of 0.80 kcal/mol, again for C3GC. The application of CP correction thus redistributes the basis-set incompleteness error more uniformly across the data set. At the DZ level, the average difference between CP-corrected and uncorrected MP2-F12D binding energies amounts to 1.38 kcal/mol, with a maximum difference of 2.76 kcal/mol for C3GC, indicating that MP2-F12D/DZ is insufficient to reliably approach the CBS limit.

Further increasing the basis set size from TZ to QZ results in substantially smaller changes in binding energies. Without CP correction, all seven complexes exhibit weaker binding, with an average change of 0.20 kcal/mol and a maximum change of 0.39 kcal/mol for C3GC. With CP correction applied, the binding energies again increase, but with a reduced average change of 0.13 kcal/mol and a maximum change of 0.23 kcal/mol. At the TZ level, the average CP and non-CP difference is 0.50 kcal/mol, with a maximum difference of 0.91 kcal/mol for C3GC, indicating that TZ is approaching, but has not yet fully reached, the CBS limit. In contrast, at the QZ level, the average CP and non-CP difference is reduced to only 0.17 kcal/mol, with a maximum difference of 0.29 kcal/mol, demonstrating that MP2-F12D/QZ is already very close to the CBS limit.

Importantly, MP2/CBS energies obtained from the extrapolation scheme are in essentially perfect agreement with CP-corrected MP2-F12D/QZ results, yielding an MAE of 0.02 kcal/mol and a MAX of 0.03 kcal/mol across the L7 data set. This level of agreement demonstrates that conventional CBS extrapolation can faithfully reproduce explicitly correlated MP2-F12D results at the basis-set limit, even for large noncovalent complexes. These findings further indicate that CBS extrapolation schemes based on finite, atom-centered Gaussian basis sets—originally developed using small model systems—remain robust and transferable to highly polarizable and electronically delocalized large complexes. On the basis of these observations, MP2-F12D in combination with a quadruple-ζ basis set is recommended as a reliable explicitly correlated approach for large noncovalent complexes when computational resources permit. More broadly, the present results confirm that MP2 correlation energies can be consistently converged to the CBS limit via extrapolation schemes, even for nanoscale noncovalent systems such as those included in the vL27 data set introduced in this work.

While our results show that MP2/CBS extrapolation using finite atom-centered Gaussian basis sets agrees well with MP2-F12 for highly polarizable and electronically delocalized systems, the ΔCCSD­(T) correlation correction is expected to be substantial in these large complexes due to the well-known tendency of MP2 to overestimate dispersion interactions.^110^ For example, the ΔCCSD­(T) correlation contributions for S12L-4a, C_60_–C_60_, C_60_@[6]­CPPA, Suman–C_60_, PClCoran–C_60_, and DClCoran–C_60_ exceed the corresponding absolute CCSD­(T)/CBS binding energies, as shown in Table S4. Therefore, a direct comparison between the composite focal-point CCSD­(T)/CBS scheme and direct CCSD­(T)/CBS extrapolation results would be valuable to further assess the reliability of using MP2-based corrections to capture basis-set effects within the focal-point framework.

### 3.2 DLPNO–CCSD­(T_1_)/CBS (VeryTightPNO, 7/8 CPS Extrapolation) Benchmark for vL27

The vL27 data set comprises 27 large noncovalent complexes ranging from 90 to 205 atoms (62–120 heavy atoms), with an average size of 136 atoms (89 heavy atoms) and a median size of 142 atoms (84 heavy atoms). The corresponding DLPNO–CCSD­(T_1_)/CBS (VeryTightPNO) results obtained using the 7/8 CPS extrapolation scheme are reported in Table 3. Relative to DLPNO–CCSD­(T1)/CBS (VeryTightPNO) without CPS extrapolation, inclusion of the 7/8 CPS scheme systematically strengthens the binding for all complexes, with differences ranging from 0.18 kcal/mol for Coran–C_60_ to 1.04 kcal/mol for C_60_@[6]­CPPA, and an average increase of 0.48 kcal/mol. For C_60_@[6]­CPPA, the reported LNO–CCSD­(T)/CBS binding energy is −41.7 ± 1.7 kcal/mol.^15^ Our DLPNO–CCSD­(T_1_)/CBS (VeryTightPNO) calculations yield −40.19 kcal/mol without CPS extrapolation and −41.23 kcal/mol with the 7/8 CPS scheme. In both cases, the DLPNO results fall within the uncertainty of the LNO–CCSD­(T)/CBS reference, while the 7/8 CPS extrapolation shifts the value closer to the center of the reported LNO benchmark range. This agreement further supports the reliability of the composite DLPNO–CCSD­(T_1_)/CBS (VeryTightPNO) protocol with 7/8 CPS extrapolation. Notably, both DLPNO–CCSD­(T_1_) and LNO–CCSD­(T) predict substantially stronger binding than the FN-DMC value of −31.1 ± 1.4 kcal/mol reported for this system.^15^

**Table 3:** Benchmark Binding Interaction Energies (kcal/mol) for the 27 Complexes (up to 205 Atoms) in the vL27 Data Set, Computed at the DLPNO–CCSD­(T_1_)/CBS Level Using the VeryTightPNO Thresholds in Conjunction with the 7/8 CPS Extrapolation Scheme^a^

vL27	DLPNO–CCSD(T_1_)/CBS
CiM-a	–41.51
CiM-c	–14.93
CiM-d	–74.16
CiM-e	–67.10
DNA-ellipticine	–39.04
S12L-3a	–38.72
S12L-4a	–39.43
S12L-5a	–43.10
S12L-5b	–29.82
S12L-7b	–27.73
S30L-5	–38.13
S30L-6	–29.03
S30L-7	–38.02
S30L-8	–43.26
S30L-13	–27.19
S30L-14	–28.96
S30L-19	–17.53
S30L-20	–21.07
S30L-22	–45.16
C_60_–C_60_	–9.90
C_60_@[6]CPPA	–41.23
Cor-C_60_	–16.58
Coran–C_60_	–11.95
Suman–C_60_	–19.21
3NSuman–C_60_	–17.44
PClCoran–C_60_	–21.23
DClCoran–C_60_	–25.00

a All binding energies were evaluated using monomer geometries extracted from the complexes without further relaxation.

#### 3.2.1 SAPT Energy Decomposition Analysis

The performance of SAPT0/jaDZ—whose accuracy in small systems benefits from favorable error cancellation^56^—and SAPT­(KS)/def2-TZVPPD combined with various *ab initio* dispersion potentials (including *ai*D3, MBD, MBDrev, and the ML-corrected variants D3-ML and MBDrev-ML) is evaluated for the vL27 data set (Figure 2). The favorable error cancellation observed for SAPT0/jaDZ in small systems^56,111,112^ does not carry over to large complexes. Indeed, SAPT0/jaDZ exhibits the largest root-mean-square error (RMSE) among the SAPT methods considered, reaching 13.2 kcal/mol in Figure 2, despite its previous use in analyzing binding in large complexes.^113^ In addition, the mean signed error (MSE) is −10.1 kcal/mol, with an MAE of 10.3 kcal/mol, indicating substantial overestimation by SAPT0/jaDZ for nearly all complexes in vL27, except S30L-19 and S30L-20. These results further confirm that the apparent robustness of SAPT0/jaDZ in small benchmark systems does not translate to larger, more complex systems. Moreover, increasing the basis set size from jaDZ to aTZ further degrades the performance of SAPT0, leading to an RMSE of 21.4 kcal/mol. This behavior indicates that SAPT0/jaDZ still relies on residual error cancellation, albeit insufficient compared to that observed in small systems. Overall, these findings highlight the necessity of incorporating intramolecular correlation effects to achieve reliable SAPT binding energies for large noncovalent complexes.

**Figure 2:**
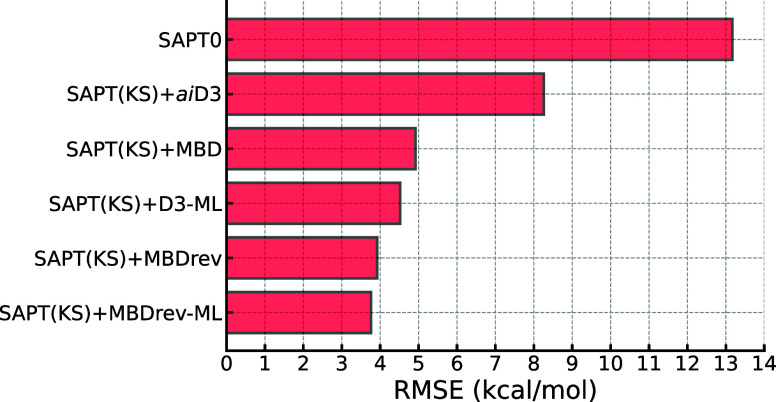
Root-mean-square errors (RMSEs, kcal/mol) for the 27 complexes in the vL27 data set computed using SAPT0 and SAPT­(KS) with various *ab initio* dispersion potentials, including *ai*D3, MBD, MBDrev, and the ML-corrected variants D3-ML and MBDrev-ML. SAPT0 calculations were carried out using the jun-cc-pVDZ basis set, whereas SAPT­(KS) calculations—based on LRC-ωPBE with system-specific ML-GDD ω tuning—were performed with the def2-TZVPPD basis set.

We first replace the problematic dispersion term in SAPT0—known to suffer from MP2-like overestimation of dispersion—with our ML-corrected D3-ML potential,^54^ leading to the SAPT0+D3-ML method.^7^ SAPT0+D3-ML/aTZ reduces the RMSE of SAPT0/aTZ to approximately one-third of its original value, lowering it from 21.4 to 7.1 kcal/mol. The RMSE can be further reduced by incorporating intramolecular correlation effects into the nondispersion terms through the use of LRC-DFT orbitals with system-specific ML-GDD ω tuning. This yields SAPT­(KS)+D3-ML, which achieves an RMSE of 4.5 kcal/mol. When SAPT­(KS) is combined with the pairwise +*ai*D3 dispersion potential,^98^ the resulting SAPT­(KS)+*ai*D3 method is known to overestimate dispersion energies in supramolecular complexes.^92,114^ This overestimation arises because the presence of numerous polarizable centers introduces screening effects that reduce the effective interaction between any two centers—an effect not captured in pairwise atom–atom models. Consequently, SAPT­(KS)+*ai*D3 exhibits the largest RMSE (8.3 kcal/mol) among all SAPT­(KS)+*ai*D variants and even underperforms relative to SAPT0+D3-ML, which corrects intramolecular correlation only in the dispersion term. In contrast, SAPT­(KS)+MBD,^99,100^ which accounts for many-body dispersion effects beyond the pairwise atom–atom approximation, reduces the RMSE to 4.9 kcal/mol. The MSE of SAPT­(KS)+MBD is −3.0 kcal/mol, indicating a systematic overestimation of binding energies by this method. We subsequently refitted the MBD parameters against SAPT2+(3)­(CCD)/aTZ dispersion energies for a broad and diverse data set of 1660 dimers, resulting in the revised +MBDrev model.^54^ SAPT­(KS)+MBDrev further reduces the RMSE to 3.9 kcal/mol and mitigates the overestimation, yielding an MSE of −1.0 kcal/mol. This improvement highlights the reliability of the revised fitting parameters and the strong transferability of the +MBDrev approach for supramolecular complexes. Finally, with the inclusion of a Δ-ML correction,^54^ SAPT­(KS)+MBDrev-ML achieves an RMSE of 3.8 kcal/mol and an MSE of only 0.06 kcal/mol, making it the most accurate SAPT-based approach in this work for describing large noncovalent complexes in the vL27 data set, as shown in Figure 2.

The most accurate SAPT-based method identified in this work, SAPT­(KS)+MBDrev-ML, is employed to analyze the binding interactions of the 27 complexes in the vL27 data set. The total interaction energy is decomposed into electrostatic, exchange, induction, and dispersion components, as summarized in Table S5. Following the classification scheme of ref 115, each complex is categorized according to its dominant binding character, as shown in Figure 1. Systems labeled **D** are dispersion-dominated, defined by |*E*_disp_/*E*_elst_| > 2, whereas those labeled **E** are electrostatics-dominated, with |*E*_disp_/*E*_elst_| < 0.5. Complexes classified as **M** exhibit mixed binding character, indicating that both dispersion and electrostatic contributions play significant roles. Among the 27 complexes in vL27, 20 are dispersion-dominated, the majority of which involve π–π stacking interactions. The remaining seven complexes display mixed binding character. Notably, even in systems featuring multiple hydrogen bonds without π–π stacking—such as CiM-a, CiM-d, S12L-5a, S12L-5b, S30L-19, and S30L-22, with |*E*_disp_/*E*_elst_| values of 0.51, 0.70, 0.91, 1.15, 1.65, and 0.51, respectively—the binding is not governed solely by electrostatics, as might be expected. Instead, dispersion interactions contribute substantially to the overall stabilization. These results highlight the critical role of accurately describing dispersion interactions for reliable modeling of binding in nanoscale systems.

Beyond absolute binding energies, accurately reproducing relative interaction strengths is equally critical in both experimental and computational studies, particularly in applications such as protein–ligand ranking in drug discovery.^116^ In this work, we examine the binding order of C_60_ with C_60_, Coran, Cor, 3NSuman, Suman, PClCoran, and DClCoran in terms of increasing interaction strength. For example, the C_60_–C_60_ and Coran–C_60_ complexes feature convex–convex and convex–concave π–π interactions, respectively, with Coran–C_60_ binding approximately 2 kcal/mol more strongly than C_60_–C_60_. However, neither SAPT0/jaDZ nor SAPT0/aTZ correctly reproduces this relative ordering. Similarly, 3NSuman–C_60_ binds about 1 kcal/mol more strongly than Cor–C_60_, yet SAPT0/jaDZ fails to capture this trend. In contrast, all SAPT­(KS)+*ai*D variants and SAPT0+D3-ML successfully reproduce these relative binding relationships. These findings demonstrate that incorporating intramolecular correlation is essential not only for achieving accurate absolute interaction energies but also for reliably predicting relative binding strengths and preserving the correct energetic ordering among competing complexes.

#### 3.2.2 Performance of Computational Methods on vL27

Figure 3 summarizes the RMSEs of all tested methods relative to the DLPNO–CCSD­(T_1_)/CBS benchmark (VeryTightPNO, 7/8 CPS extrapolation) for the vL27 data set, including MP2-based methods, DFT approaches, composite electronic-structure methods, semiempirical methods, and MLPs. Tables S6–S14 report the individual binding energies for each complex, together with the corresponding MAE, RMSE, MAX, and MSE values across all computational methods considered for the vL27 data set.

**Figure 3:**
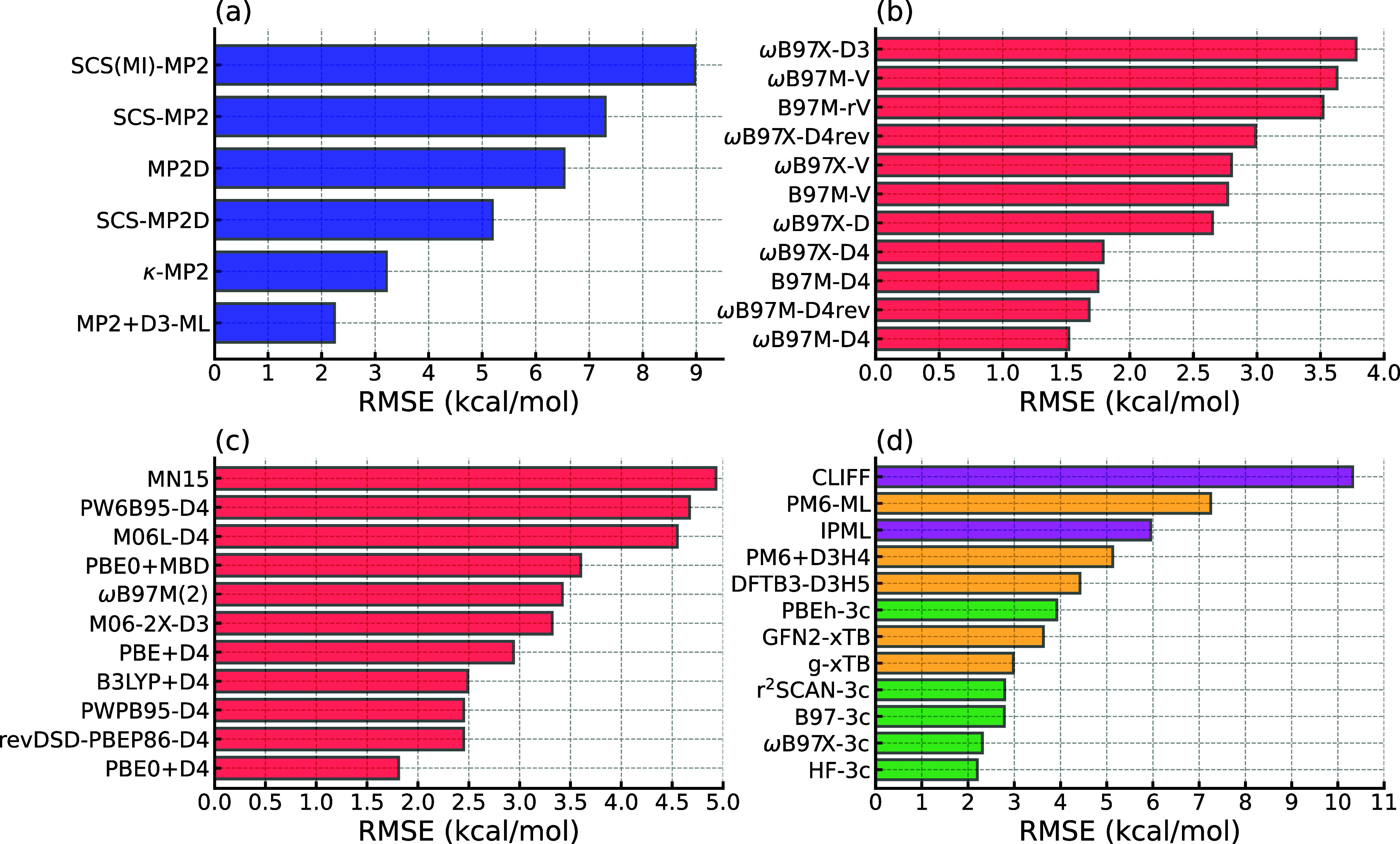
Root-mean-square errors (RMSEs, kcal/mol) for the 27 complexes (up to 205 atoms) in the vL27 data set relative to the DLPNO–CCSD­(T_1_)/CBS benchmark (VeryTightPNO, 7/8 CPS extrapolation). Results are shown for (a) MP2-based methods (blue), (b,c) DFT methods (red), and (d) composite electronic-structure methods (green), semiempirical methods (orange), and machine learning potentials (purple). All MP2 calculations, except MP2+D3-ML and κ-MP2, were extrapolated to the CBS limit. MP2+D3-ML and κ-MP2 were computed with the aTZ basis set. All DFT calculations employed the def2-TZVPPD basis set, except for the three double-hybrid functionals PWPB95-D4, revDSD-PBEP86-D4, and ωB97M(2), for which results were extrapolated to the CBS limit. BSSE corrections were applied to all MP2 and DFT results using the counterpoise method. MP2/CBS is excluded from the figure due to its exceptionally large RMSE (19.79 kcal/mol). IPML results are reported for 23 complexes because the method is not available for charged systems or complexes containing Cl or S atoms. CLIFF results are available for 15 complexes, as the method is not applicable to charged systems and failed to converge for 11 complexes.

As expected, conventional MP2 substantially overestimates binding energies for all complexes in the vL27 data set, including hydrogen-bonded systems. It yields an RMSE of 19.8 kcal/mol and a MAX of 49.7 kcal/mol for C_60_@[6]­CPPA, making it the least accurate method examined. All modified MP2 variants—spin-component-scaled (SCS), dispersion-corrected, regularized, and ML-enhanced approaches—show significant improvement over standard MP2. Although SCS­(MI)-MP2 was specifically parametrized for NCIs, it performs worst among the improved variants, with an RMSE of 9.0 kcal/mol and an MSE of −5.5 kcal/mol, indicating substantial overestimation of binding energies. This outcome underscores the limited transferability of fixed SCS coefficients optimized for small benchmark systems. In contrast, the original SCS-MP2 method, whose scaling coefficients were fitted to representative reaction energies, performs better, achieving an RMSE of 7.3 kcal/mol and an MSE of −1.6 kcal/mol. Dispersion-corrected MP2D and its SCS variant (SCS-MP2D) further improve accuracy, yielding RMSEs of 6.5 and 5.2 kcal/mol, respectively; however, they still exhibit substantial MSEs of −4.8 and −3.9 kcal/mol, indicating persistent overestimation. The regularized κ-MP2 method (κ = 1.1) demonstrates strong transferability to large complexes and performs even better, with an RMSE of 3.2 kcal/mol and an MSE of −1.0 kcal/mol.

Among all MP2-based approaches, our MP2+D3-ML method delivers the best overall performance, with an RMSE of 2.2 kcal/mol and a MAX of 4.0 kcal/mol (from S30L-8). In contrast, the maximum errors of the other MP2-based methods exceed 8 kcal/mol. The MSE of MP2+D3-ML is only 0.6 kcal/mol, indicating a slight underestimation, in contrast to the substantial overestimation observed for other MP2-based variants. MP2+D3-ML therefore exhibits excellent transferability to large-scale NCIs, including complexes containing up to 205 atoms in vL27, outperforming SCS-MP2, SCS­(MI)-MP2, MP2D, and SCS-MP2D by at least a factor of 2 in both MAE and MAX (Figure 3a). Although κ-MP2 ranks second among the MP2 variants (RMSE = 3.2 kcal/mol), its MAX remains relatively large (8.9 kcal/mol for C_60_@[6]­CPPA). Notably, all MP2-based methods except MP2+D3-ML and κ-MP2 exhibit particularly large errors (>10 kcal/mol) for C_60_@[6]­CPPA, with overestimations of approximately 50, 24, 18, 17, and 13 kcal/mol for MP2, SCS-MP2, SCS­(MI)-MP2, MP2D, and SCS-MP2D, respectively. These substantial deviations likely stem from the strong confinement and pronounced many-body effects in C_60_@[6]­CPPA, which are inadequately described by these approaches.^7,55^ In contrast, MP2+D3-ML shows only a minor underestimation for this complex (3.7 kcal/mol). Its superior performance can be attributed to the integration of a data-driven ML model within the *ai*D potential, which enhances the treatment of many-body dispersion effects.

Regarding relative binding trends, conventional MP2 incorrectly predicts the ordering of C_60_–C_60_ vs Coran–C_60_ and Cor–C_60_ vs 3NSuman–C_60_. SCS-MP2, SCS­(MI)-MP2, MP2D, and SCS-MP2D also fail to reproduce the latter trend. In contrast, both MP2+D3-ML and κ-MP2 correctly predict the binding orderings, further demonstrating their reliability in describing NCIs in large noncovalent complexes.

Figure 3b,c present the performance of DFT methods on the vL27 data set. Among the four VV10-based Head-Gordon functionals shown in Figure 3b, namely ωB97X-V, ωB97M-V, B97M-V, and B97M-rV, B97M-V delivers the best overall performance, with an RMSE of 2.8 kcal/mol, an MSE of −0.6 kcal/mol, and the lowest MAX of 5.5 kcal/mol. In B97M-rV, the original VV10 kernel is replaced by the simplified and computationally more efficient rVV10 kernel,^60^ which enables applications to systems containing thousands of atoms.^117^ Although B97M-rV has been reported to provide slightly improved accuracy for small-molecule NCIs,^60^ its behavior differs for the large complexes in vL27. The rVV10 kernel systematically strengthens binding across all 27 complexes, leading to an average overbinding of 1.5 kcal/mol relative to B97M-V. As a result, B97M-rV performs somewhat worse, with an RMSE of 3.5 kcal/mol and the largest MAX (7.5 kcal/mol) among the four VV10-based functionals. For the range-separated hybrid variants, ωB97X-V performs comparably to B97M-V, yielding an RMSE of 2.8 kcal/mol. In contrast, ωB97M-V shows the weakest performance among the VV10-based functionals, with an RMSE of 3.6 kcal/mol, despite being the level of theory adopted in the recently proposed Open Molecules 2025 (OMol25) data set.^118^

Because the VV10 nonlocal correlation kernel accounts only for two-body dispersion effects,^119^ VV10-based functionals are expected to overestimate binding in large complexes where many-body dispersion contributions become important. This trend is evident in the vL27 data set: overbinding occurs in 24, 16, 13, and 17 complexes for ωB97M-V, ωB97X-V, B97M-V, and B97M-rV, respectively. Improved performance can be achieved by incorporating three-body dispersion effects, as in DFT-D3 corrections.^7,61,120,121^ Rather than adding a three-body term on top of VV10, several newer functionals replace the VV10 kernel entirely with the D4 dispersion correction, which includes three-body contributions. These include ωB97X-D4,^61^ ωB97M-D4,^61^ ωB97X-D4rev,^62^ ωB97M-D4rev,^63^ and B97M-D4.^61^ As shown in Figure 3b, replacing the VV10 nonlocal correlation with D4 generally leads to substantial improvements in the parent functionals. The only exception is ωB97X-D4rev, for which the RMSE slightly increases from 2.8 kcal/mol (ωB97X-V) to 3.0 kcal/mol. Among all DFT methods examined, ωB97M-D4 delivers the best overall performance, achieving an RMSE of 1.5 kcal/mol. ωB97M-D4 shows a slight underestimation, with an MSE of 1.2 kcal/mol, in contrast to the overestimation observed for ωB97M-V, which has an MSE of −2.8 kcal/mol. For nonhybrid functionals, B97M-D4 performs best, with an RMSE of 1.8 kcal/mol. Both ωB97X-D4 and ωB97M-D4rev also exhibit strong performance, with RMSEs of 1.8 and 1.7 kcal/mol, respectively. In addition, ωB97M-D4rev achieves the lowest MSE among the DFT methods, at 0.2 kcal/mol, indicating only a slight underestimation. Notably, the older ωB97X-D functional (RMSE = 2.7 kcal/mol) still outperforms the more recent ωB97X-V and ωB97M-V variants. In contrast, updating ωB97X-D with the D3 dispersion correction to form ωB97X-D3 leads to the poorest performance among the B97-type functionals tested, with an RMSE of 3.8 kcal/mol and a MAX of 10.7 kcal/mol (for the C_60_@[6]­CPPA complex), as shown in Figure 3b. Despite these differences in absolute accuracy, all 11 B97-type functionals successfully reproduce the correct relative binding trends for C_60_ with the seven complexes discussed above.

The four dispersion-corrected functionals incorporating D4 or MBD corrections—PBE0+D4, PBE+D4, PBE0+MBD, and B3LYP+D4—yield RMSEs of 1.8, 2.9, 3.6, and 2.5 kcal/mol, respectively. These results are consistent with our previous finding that PBE0+D4 is a reliable choice for investigating NCIs in large complexes.^7^ Among all computational methods tested, PBE0+D4 achieves the lowest MAX (3.2 kcal/mol), observed for the C_60_@[6]­CPPA complex. For all 27 complexes, PBE0+D4 predicts consistently stronger binding than PBE+D4, resulting in substantially improved overall accuracy; specifically, PBE0+D4 is approximately 1.5 times more accurate in terms of RMSE. Moreover, PBE0+D4 outperforms PBE0+MBD by roughly a factor of 2, consistent with previous findings that DFT+D4 provides better agreement with local CCSD­(T)/CBS benchmarks than DFT+MBD.^15^ When the D4 correction is combined with the widely used hybrid functional B3LYP, B3LYP+D4 exhibits slightly worse performance than PBE0+D4. Regarding relative binding trends, B3LYP+D4, PBE0+D4, and PBE+D4 incorrectly predict the ordering of Cor–C_60_ vs 3NSuman–C_60_. In addition, PBE0+MBD introduces a new error in the relative binding sequence: while benchmark results indicate that PClCoran–C_60_ binds approximately 2 kcal/mol more strongly than Suman–C_60_, PBE0+MBD predicts the opposite ordering, with Suman–C_60_ binding 0.6 kcal/mol more strongly than PClCoran–C_60_.

The four Minnesota functionals—PW6B95-D4, M06–2X-D3, M06-L-D4, and MN15—yield RMSEs of 4.7, 3.3, 4.6, and 4.9 kcal/mol, respectively. These results suggest that newer generations of Minnesota functionals do not necessarily provide improved accuracy. All four functionals show noticeable deterioration in performance for large noncovalent complexes. Among the tested DFT methods, MN15 exhibits the largest MAX of 11.6 kcal/mol, followed by PW6B95-D4 with a MAX of 10.9 kcal/mol; in both cases, the dominant error arises from substantial underestimation of C_60_@[6]­CPPA. This finding contrasts with ref 122, which identified PW6B95-D4 as the best-performing functional for large noncovalent complexes when benchmarked against CIM-DLPNO–CCSD­(T). The discrepancy highlights the critical importance of employing reliable reference methods when evaluating lower-cost computational approaches. In addition, PW6B95-D4, M06–2X-D3, and MN15 fail to reproduce the correct binding order of PClCoran–C_60_ vs Suman–C_60_. In contrast, M06-L-D4 successfully captures the binding trends for those challenging C_60_-containing complexes.

The double-hybrid functionals PWPB95-D4 and revDSD-PBEP86-D4 have been identified as among the most reliable methods for describing ion–π interactions in the ION19 data set.^123^ Similarly, ωB97M(2) has been recommended as one of the best Rung 5 density functionals.^79^ For the vL27 data set, PWPB95-D4, revDSD-PBEP86-D4, and ωB97M(2) yield RMSEs of 3.1, 3.2, and 2.8 kcal/mol, respectively, with MAX values of 5.5 kcal/mol (S30L-8), 7.8 kcal/mol (CiM-e), and 7.1 kcal/mol (S12L-4a) when using the def2-TZVPPD basis set. Since the use of a quadruple-ζ basis set, or extrapolation to the CBS limit, is recommended for double-hybrid functionals due to their MP2 correlation component, the def2-TZVPP and def2-QZVPP basis sets, as well as CBS-extrapolated results based on these, were employed for these three functionals. Compared with the def2-TZVPPD results, PWPB95-D4 and revDSD-PBEP86-D4 using def2-TZVPP show slightly increased RMSEs of 3.7 and 3.9 kcal/mol, respectively, whereas ωB97M(2) exhibits improved performance, with its RMSE reduced to 2.4 kcal/mol when using def2-TZVPP. When the quadruple-ζ def2-QZVPP basis set is used, even without diffuse functions, PWPB95-D4 and revDSD-PBEP86-D4 show modest improvements, yielding RMSEs of 2.8 and 2.9 kcal/mol, respectively. Their performance further improves upon extrapolation to the CBS limit, with RMSEs of 2.5 kcal/mol for both functionals. In contrast, the performance of ωB97M(2) deteriorates with increasing basis set size, with an RMSE of 3.1 kcal/mol at the def2-QZVPP level and 3.4 kcal/mol at the CBS limit. Thus, its best performance is obtained with the smaller def2-TZVPP basis set used in this work, which is not consistent with the expected basis-set convergence behavior of a double-hybrid functional. Overall, PWPB95-D4 and revDSD-PBEP86-D4 exhibit improved performance with larger basis sets, whereas ωB97M(2) does not follow this trend. The CBS-limit results for these three double-hybrid functionals are shown in Figure 3c.

However, even at the CBS limit, PWPB95-D4 and revDSD-PBEP86-D4 perform approximately 1 kcal/mol worse than the best-performing DFT functional for NCIs in the vL27 data set, ωB97M-D4, which achieves an RMSE of 1.5 kcal/mol. These results indicate that ascending to the highest rung of Perdew’s Jacob’s Ladder, represented by double-hybrid functionals, does not necessarily guarantee superior accuracy, despite the inclusion of second-order perturbative correlation at substantially higher computational cost. Compared with other MP2-based methods, PWPB95-D4 and revDSD-PBEP86-D4 at the CBS limit outperform all MP2-based variants except MP2+D3-ML, which achieves an RMSE of 2.2 kcal/mol. Regarding relative binding trends, PWPB95-D4 consistently predicts 3NSuman–C_60_ to bind more strongly than Cor–C_60_ by about 1 kcal/mol across all basis sets. In contrast, revDSD-PBEP86-D4 reverses this ordering when using the def2-TZVPPD, def2-TZVPP, and def2-QZVPP basis sets. Only at the CBS limit does revDSD-PBEP86-D4 recover the correct ordering, and even then by a marginal 0.01 kcal/mol. This inconsistency underscores the limited transferability of fixed SCS coefficients across different chemical systems. Additionally, ωB97M(2) reproduces the correct binding order of 3NSuman–C_60_ and Cor–C_60_ when using the smaller def2-TZVPP and def2-TZVPPD basis sets, but predicts an incorrect ordering with the def2-QZVPP basis set and at the CBS limit, suggesting that extrapolation of ωB97M(2) results to the CBS limit may not be reliable. Overall, PWPB95-D4 emerges as the most reliable double-hybrid functional for describing NCIs in large noncovalent complexes, with an RMSE of 2.5 kcal/mol and a consistent, moderate underestimation of binding energies (MSE = 2.2 kcal/mol).

Among the five composite electronic-structure methods, HF-3c, B97–3c, *r* ^2^SCAN-3c, and ωB97X-3c achieve RMSEs below 3 kcal/mol for the vL27 data set, with values of 2.2, 2.8, 2.8, and 2.3 kcal/mol, respectively (Figure 3d). In general, these composite methods outperform semiempirical approaches and MLPs, with the notable exception of PBEh-3c. PBEh-3c yields an RMSE of 3.9 kcal/mol, underperforming relative to g-xTB, GFN2-xTB, and DFTB3-D3H5, and thus stands out as the only composite electronic-structure method in this set that is not well suited for large noncovalent complexes. Despite being the earliest member of the 3c family and employing the minimal MINIX basis set, HF-3c delivers the best overall performance among the five 3c methods, with the lowest RMSE (2.2 kcal/mol), the smallest MAX (4.8 kcal/mol for CiM-d), and an MSE of only −0.1 kcal/mol. The most recent 3c method, ωB97X-3c, ranks second, achieving an RMSE of 2.3 kcal/mol, albeit requiring a double-ζ basis set. For applications favoring nonhybrid functionals, B97–3c and *r* ^2^SCAN-3c are recommended; both provide RMSEs of 2.8 kcal/mol, though at the cost of employing triple-ζ basis sets. Interestingly, among the five composite electronic-structure methods, only ωB97X-3c fails to reproduce the correct binding order of Cor–C_60_ vs 3NSuman–C_60_.

For the semiempirical methods, both g-xTB and GFN2-xTB outperform PM6-D3H4 and DFTB3-D3H5, yielding RMSEs of 3.0 and 3.6 kcal/mol, respectively, compared to 5.1 and 4.4 kcal/mol for PM6-D3H4 and DFTB3-D3H5. The recently proposed ML-corrected PM6 method (PM6-ML) has been reported to surpass standalone semiempirical approaches and MLPs.^88^ However, on the vL27 data set, its performance deteriorates relative to PM6-D3H4, with an RMSE of 7.3 kcal/mol. Among the semiempirical approaches, g-xTB performs best (RMSE = 3.0 kcal/mol), improving upon its predecessor GFN2-xTB (RMSE = 3.6 kcal/mol). In terms of relative binding trends, both g-xTB and GFN2-xTB incorrectly predict the ordering of C_60_–C_60_ vs Coran–C_60_. Moreover, PM6-ML substantially degrades the relative binding trends obtained with PM6-D3H4, incorrectly predicting the orderings of both C_60_–C_60_ vs Coran–C_60_ and Suman–C_60_ vs 3NSuman–C_60_. Notably, PM6-ML predicts C_60_–C_60_ to bind 8.6 kcal/mol more strongly than Coran–C_60_, whereas the reference data indicate that Coran–C_60_ binds 2.1 kcal/mol more strongly than C_60_–C_60_. In fact, PM6-ML is the only method tested that fails to reproduce the correct binding order of Suman–C_60_ vs 3NSuman–C_60_. These results highlight the challenge of achieving robust transferability with ML approaches across chemically diverse systems of varying size and interaction type, even when such models are combined with semiempirical methods.

The two MLPs, IPML and CLIFF, are unable to evaluate all 27 complexes in the vL27 data set. IPML lacks parameters for Cl and S and does not support charged systems, limiting its applicability to 23 complexes. CLIFF suffers from convergence issues and similarly lacks support for charged species, restricting its evaluation to only 15 complexes. For the subsets of systems they can treat, IPML and CLIFF yield relatively large RMSEs of 6.0 and 10.3 kcal/mol, respectively, outperforming only SAPT0/jaDZ, SAPT0/aTZ, and conventional MP2. Their accuracy deteriorates markedly with increasing system size, indicating limited transferability to larger complexes and rendering them less reliable than other low-cost computational approaches examined in this work. Interestingly, for the accessible C_60_-containing complexes that present challenging binding trends, IPML successfully reproduces the correct relative ordering, despite exhibiting large absolute errors in the binding energies.

## 4 Conclusions

This work introduces a new benchmark data set for large noncovalent complexes, vL27, comprising 27 systems with sizes up to 205 atoms and featuring highly polarizable, electronically delocalized structures. The data set spans a diverse range of binding motifs, including hydrogen bonding, π–π stacking, long-range chain interactions, convex–concave π contacts, confinement-induced interactions, and pronounced many-body dispersion effects. Building upon our previously introduced vL11 data set (11 systems, up to 174 atoms),^7^ vL27 incorporates 16 additional large complexes, extending the system size beyond the 200-atom regime. SAPT analysis reveals that most systems are either dispersion-dominated or exhibit mixed interaction character, even in complexes involving hydrogen bonds without explicit π–π stacking. These results highlight the central role of dispersion and many-body effects in stabilizing large noncovalent complexes.

The vL27 systems pose significant challenges due not only to intricate π–π interaction patterns but also to pronounced many-body and confinement effects, both of which complicate the accurate description of NCIs. Reference binding energies for vL27 were computed at the DLPNO–CCSD­(T_1_)/CBS (VeryTightPNO) level using 7/8 CPS extrapolation, whereas the previously introduced vL11 data set was evaluated at the same level without CPS extrapolation.^7^ The present protocol reduces errors associated with local approximations and, as demonstrated here, provides benchmark-quality reference energies for large noncovalent complexes, even at the two-hundred-atom scale where canonical CCSD­(T)/CBS calculations are computationally prohibitive. Furthermore, we demonstrate that the CPS extrapolation scheme remains reliable for nanoscale noncovalent systems when VeryTightPNO thresholds are employed The accuracy of the MP2 basis-set extrapolation was also validated through direct comparison with MP2-F12 calculations using quadruple-ζ basis sets. The close agreement between MP2/CBS and MP2-F12 results confirms that conventional CBS extrapolation remains robust for large noncovalent complexes.

The vL27 data set thus represents a new frontier for electronic-structure theory, enabling the systematic evaluation of complex quantum-mechanical effects in large, finite, nonperiodic nanoscale systems. Benchmarking a broad range of lower-cost methods—including MP2-based approaches, DFT functionals, composite electronic-structure methods, semiempirical methods, and MLPs—provides valuable guidance for both method selection and future development. In particular, vL27 enables assessment of whether methods that perform well for smaller benchmark systems retain their accuracy and transferability for large complexes characterized by intricate many-body dispersion and confinement effects. Notably, several methods are evaluated here for the first time on large noncovalent complexes in vL27, including SAPT-based approaches, double-hybrid functionals, Head-Gordon functionals with D4 dispersion in place of VV10, and κ-MP2, among others, which were not assessed in our previously introduced vL11 data set.^7^

Among SAPT approaches, SAPT­(KS)+MBDrev-ML (including refitted MBD parameters and an additional ML correction) performs best, with an RMSE of 3.8 kcal/mol and a MAX of 8.5 kcal/mol. For MP2-based methods, MP2+D3-ML achieves the highest accuracy (RMSE = 2.2 kcal/mol; MAX = 4.0 kcal/mol), followed by κ-MP2 (RMSE = 3.2 kcal/mol), although the latter exhibits a relatively large MAX (8.9 kcal/mol). Within DFT, the best-performing nonhybrid functional is B97M-D4 (RMSE = 1.8 kcal/mol; MAX = 4.1 kcal/mol), while the top-performing hybrid functional is ωB97M-D4 (RMSE = 1.5 kcal/mol; MAX = 3.7 kcal/mol). PBE0+D4, previously recommended for large noncovalent complexes in vL11,^7^ performs reliably on vL27 (RMSE = 1.8 kcal/mol) and yields the lowest MAX among all tested methods (3.2 kcal/mol). Notably, PBE+D4 stands out among nonhybrid, non-VV10 functionals (RMSE = 2.9 kcal/mol; MAX = 5.6 kcal/mol). Overall, these results underscore the importance of incorporating Hartree–Fock exchange and/or nonlocal correlation (e.g., the VV10 kernel) for accurate treatment of large noncovalent complexes.

Among composite electronic-structure methods, HF-3c performs best (RMSE = 2.2 kcal/mol), followed by ωB97X-3c (2.3 kcal/mol), B97–3c (2.8 kcal/mol), and *r* ^2^SCAN-3c (2.8 kcal/mol). For semiempirical methods, the latest generation g-xTB achieves the best performance (RMSE = 3.0 kcal/mol; MAX = 7.6 kcal/mol). In contrast, the MLPs IPML and CLIFF exhibit significantly larger errors (RMSEs of 6.0 and 10.3 kcal/mol, respectively), reflecting limited transferability to large complexes. Evaluation of more advanced MLPs such as AIMNet2 ^124^ and UMA^125^ for vL27 is currently underway in our group.

With respect to relative binding trends—specifically, the ordering of C_60_ with C_60_, coronene, corannulene, sumanene, triazasumanene, pentachlorocorannulene, and decachlorocorannulene—PBE0+D4, PBE+D4, ωB97X-3c, and g-xTB fail to reproduce the correct energetic sequence, despite performing well for absolute binding energies as discussed above. In contrast, SAPT­(KS)+MBDrev-ML, MP2+D3-ML, κ-MP2, B97M-D4, ωB97M-D4, HF-3c, B97–3c, and r^2^SCAN-3c successfully capture the correct binding order. Considering both absolute interaction energies and relative trends, MP2+D3-ML, B97M-D4, ωB97M-D4, and HF-3c emerge as the most reliable and transferable approaches for studying NCIs in large complexes. Notably, this conclusion differs slightly from that of our previous vL11 study,^7^ which employed the DLPNO–CCSD­(T_1_)/CBS (VeryTightPNO) level without CPS extrapolation and recommended MP2+D3-ML, PBE0+D4, and ωB97X-3c based primarily on absolute binding performance. When both absolute energies and relative trends are taken into account, MP2+D3-ML remains a consistently recommended method, alongside the Head-Gordon functionals with D4 dispersion (B97M-D4 and ωB97M-D4) and the low-cost HF-3c approach.

Importantly, HF-3c provides an attractive prescreening tool for large sets of candidate complexes prior to higher-accuracy refinement using methods such as MP2+D3-ML or advanced DFT functionals (B97M-D4 and ωB97M-D4). These four methods therefore represent practical and accurate choices for investigating realistic biomolecular complexes, host–guest systems, and supramolecular assemblies governed by NCIs when CCSD­(T)/CBS-level treatments are computationally infeasible. Together, the vL27 data set introduced in this work, along with our previously reported L14 ^7^ and CC3@7 data sets,^8^ establishes a rigorous benchmark framework for probing complex chemical environments at the nanoscale and for guiding the development, calibration, and validation of computationally efficient methods—including MLPs—for large, chemically realistic systems.

## Supplementary Material





## Notes

See the Supporting Information for the binding energies of the computational methods used in this work (PDF)
Along with the Cartesian coordinates for the 27 complexes in vL27 (ZIP)
