# Supplementary material for: Benchmarking Nanoscale Noncovalent Complexes at the Two-Hundred-Atom Scale with Converged Local CCSD(T)
Source: J Phys Chem A. 2026 May 23;130(22):4136–51. doi: 10.1021/acs.jpca.6c01097 (PMC13244456; doi:10.1021/acs.jpca.6c01097)
Supplement: Supplementary file 1 [file jp6c01097_si_001.pdf]

Supporting Information for
"Benchmarking Nanoscale Noncovalent Complexes at the Two-Hundred-Atom Scale with Converged Local CCSD(T)"

Ka Un Lao*
Department of Chemistry, Virginia Commonwealth University, Richmond, VA 23284 USA

TABLE S1: Binding correlation energies (kcal/mol) for the L14 dataset, calculated using canonical CCSD(T)/def2-SVP and various levels of DLPNO-CCSD(T_1)/def2-SVP. Mean absolute error (MAE) and maximum absolute error (MAX) relative to canonical CCSD(T) are also provided.

L14	canonical CCSD(T)	DLPNO-CCSD(T_1) TightPNO	DLPNO-CCSD(T_1) VeryTightPNO	DLPNO-CCSD(T_1) TightPNO (6/7 CPS)	DLPNO-CCSD(T_1) VeryTightPNO (7/8 CPS)
C2C2PD	−27.42	−28.95	−26.77	−29.50	−27.05
C3A	−18.22	−19.90	−17.99	−20.45	−18.29
C3GC	−31.91	−34.35	−31.52	−35.14	−31.97
CBH	−12.36	−12.45	−12.07	−12.60	−12.19
GCGC	−16.81	−17.13	−16.47	−17.62	−16.71
GGG	−7.14	−7.31	−7.08	−7.45	−7.19
PHE	−4.26	−4.23	−4.04	−4.48	−4.17
2a	−28.55	−29.90	−27.90	−30.69	−28.39
2b	−22.57	−23.74	−22.24	−24.22	−22.52
3b	−27.01	−28.35	−26.57	−29.25	−27.05
S8-1	−27.32	−28.31	−26.93	−28.97	−27.33
S8-2	−23.26	−24.22	−22.71	−24.86	−23.12
Da1	−29.08	−30.40	−28.58	−30.94	−28.91
Da2	−26.28	−27.59	−25.84	−28.09	−26.15
MAE	–	1.05	0.39	1.58	0.11
MAX	–	2.44	0.65	3.23	0.37

TABLE S2: Binding energies (kcal/mol) for the L7 dataset computed using MP2/CBS and MP2-F12 with cc-pV*n*Z-F12 basis sets (*n* = D, T, Q), denoted as DZ, TZ, and QZ, respectively. Both uncorrected and CP-corrected MP2-F12 binding energies are reported. Mean absolute error (MAE) and maximum absolute error (MAX), relative to the MP2/CBS, reference are also provided.

	MP2 CBS	MP2-F12 DZ	MP2-F12 (CP) DZ	MP2-F12 TZ	MP2-F12 (CP) TZ	MP2-F12 QZ	MP2-F12 (CP) QZ
C2C2PD	−38.07	−39.21	−37.36	−38.69	−37.91	−38.32	−38.08
C3A	−27.04	−27.93	−26.69	−27.43	−26.94	−27.22	−27.06
C3GC	−45.26	−46.80	−44.26	−45.95	−45.05	–	–
CBH	−11.84	−12.02	−11.57	−11.89	−11.75	−11.87	−11.82
GCGC	−18.95	−19.61	−18.25	−19.39	−18.76	−19.15	−18.95
GGG	−4.50	−4.76	−4.22	−4.67	−4.41	−4.58	−4.49
PHE	−26.45	−26.82	−26.09	−26.66	−26.40	−26.57	−26.48
MAE	–	0.72	0.53	0.37	0.13	0.14	0.02
MAX	–	1.54	1.00	0.69	0.21	0.25	0.02

* email: laoku@vcu.edu

TABLE S3: Binding energies (kcal/mol) for the L7 dataset computed using MP2/CBS and MP2-F12D with cc-pVnZ-F12 basis sets (n = D, T, Q), denoted as DZ, TZ, and QZ, respectively. Both uncorrected and CP-corrected MP2-F12D binding energies are reported. Mean absolute error (MAE) and maximum absolute error (MAX), relative to the MP2/CBS, reference are also provided.

	MP2 CBS	MP2-F12D DZ	MP2-F12D (CP) DZ	MP2-F12D TZ	MP2-F12D (CP) TZ	MP2-F12D QZ	MP2-F12D (CP) QZ
C2C2PD	−38.07	−39.39	−37.36	−38.71	−37.91	−38.33	−38.08
C3A	−27.04	−28.05	−26.48	−27.44	−26.94	−27.23	−27.06
C3GC	−45.26	−47.02	−44.26	−45.96	−45.06	−45.58	−45.29
CBH	−11.84	−12.08	−11.57	−11.90	−11.75	−11.88	−11.82
GCGC	−18.95	−19.68	−18.24	−19.39	−18.76	−19.16	−18.95
GGG	−4.50	−4.79	−4.22	−4.67	−4.41	−4.58	−4.49
PHE	−26.45	−26.83	−26.06	−26.67	−26.40	−26.57	−26.47
MAE		0.82	0.56	0.38	0.13	0.17	0.02
MAX		1.76	1.00	0.70	0.20	0.32	0.03

TABLE S4: The table summarizes the binding energy components $E^{\mathrm{HF/CBS}}$, $E_{\mathrm{corr}}^{\mathrm{MP2/CBS}}$, $\Delta E_{\mathrm{corr}}^{\mathrm{CCSD(T)/small}}$, and $\Delta\Delta E_{\mathrm{corr}}^{\mathrm{CCSD(T)}}$ that are combined to obtain $E^{\mathrm{CCSD(T)/CBS}}$ for the 27 complexes in the vL27 dataset. All values are computed at the DLPNO-CCSD(T_1)/CBS level using VeryTightPNO thresholds and the 7/8 CPS extrapolation scheme. The level of theory for each component is detailed in Table II.

vL27	$E^{\mathrm{HF/CBS}}$	$E_{\mathrm{corr}}^{\mathrm{MP2/CBS}}$	$\Delta E_{\mathrm{corr}}^{\mathrm{CCSD(T)/small}}$	$\Delta\Delta E_{\mathrm{corr}}^{\mathrm{CCSD(T)}}$	$E^{\mathrm{CCSD(T)/CBS}}$
CiM-a	−20.88	−21.32	0.59	0.10	−41.51
CiM-c	4.72	−24.45	3.81	0.99	−14.93
CiM-d	−31.27	−44.93	1.09	0.95	−74.16
CiM-e	−0.48	−74.24	5.56	2.05	−67.10
DNA-ellipticine	25.17	−80.77	12.27	4.29	−39.04
S12L-3a	23.60	−80.90	14.80	3.78	−38.72
S12L-4a	34.66	−114.51	34.47	5.94	−39.43
S12L-5a	−10.67	−37.94	4.42	1.08	−43.10
S12L-5b	−0.36	−35.96	5.45	1.05	−29.82
S12L-7b	10.44	−43.22	4.20	0.85	−27.73
S30L-5	18.49	−78.82	18.08	4.11	−38.13
S30L-6	24.86	−75.39	17.47	4.04	−29.03
S30L-7	40.02	−107.63	22.76	6.83	−38.02
S30L-8	43.53	−119.79	25.43	7.57	−43.26
S30L-13	8.16	−43.25	6.22	1.69	−27.19
S30L-14	13.12	−53.23	8.53	2.63	−28.96
S30L-19	2.35	−21.06	1.09	0.09	−17.53
S30L-20	8.68	−31.60	1.70	0.15	−21.07
S30L-22	−26.08	−19.75	0.53	0.15	−45.16
C_{60}–C_{60}	9.10	−29.32	9.37	0.95	−9.90
C_{60}@[6]CPPA	56.35	−147.27	40.73	8.95	−41.23
Cor–C_{60}	15.61	−50.72	16.07	2.46	−16.58
Coran–C_{60}	2.39	−21.38	6.31	0.73	−11.95
Suman–C_{60}	22.35	−62.21	17.60	3.05	−19.21
3NSuman–C_{60}	20.88	−55.66	14.98	2.36	−17.44
PClCoran–C_{60}	24.13	−68.80	19.32	4.12	−21.23
DClCoran–C_{60}	26.27	−79.30	23.16	4.86	−25.00

TABLE S5: Energy decomposition analysis of 27 complexes in the vL27 dataset. The total binding energy is partitioned into electrostatic, exchange, induction, and dispersion components (kcal/mol) using SAPT(KS)+MBDrev-ML/def2-TZVPPD with LRC-ωPBE and system-specific ω values determined via ML-GDD tuning. Each complex is classified according to its dominant binding character based on SAPT(KS)+MBDrev-ML/def2-TZVPPD results: **D** denotes dispersion-dominated systems ($|E_{\rm disp}/E_{\rm elst}| > 2$), **E** denotes electrostatics-dominated systems ($|E_{\rm disp}/E_{\rm elst}| < 0.5$), and **M** denotes mixed binding character, where both electrostatic and dispersion contributions are significant.

vL27	$E_{\rm elst}$	$E_{\rm exch}$	$E_{\rm ind}$	$E_{\rm disp}$	$E_{\rm total}$	$\|E_{\rm disp}/E_{\rm elst}\|$	Binding Character
CiM-a	−65.40	85.13	−21.69	−33.41	−35.36	0.51	M
CiM-c	−6.29	13.92	−1.79	−20.40	−14.56	3.25	D
CiM-d	−99.65	138.23	−36.90	−69.70	−68.02	0.70	M
CiM-e	−87.40	146.1	−28.04	−89.27	−58.61	1.02	M
DNA-ellipticine	−21.52	57.13	−6.52	−68.77	−39.68	3.20	D
S12L-3a	−22.24	56.70	−7.85	−66.53	−39.93	2.99	D
S12L-4a	−26.65	66.37	−7.10	−73.20	−40.58	2.75	D
S12L-5a	−48.19	69.23	−19.38	−44.03	−42.36	0.91	M
S12L-5b	−33.25	54.51	−11.94	−38.16	−28.83	1.15	M
S12L-7b	−10.05	28.95	−3.26	−44.72	−29.08	4.45	D
S30L-5	−27.02	58.12	−9.34	−62.32	−40.56	2.31	D
S30L-6	−28.10	66.84	−11.84	−62.50	−35.60	2.22	D
S30L-7	−21.33	65.77	−5.92	−83.24	−44.73	3.90	D
S30L-8	−21.58	69.42	−6.17	−92.50	−50.83	4.29	D
S30L-13	−14.56	29.99	−3.72	−39.07	−27.36	2.68	D
S30L-14	−17.86	38.90	−4.51	−46.75	−30.22	2.62	D
S30L-19	−14.47	26.69	−4.25	−23.88	−15.90	1.65	M
S30L-20	−17.42	38.44	−4.99	−36.01	−19.98	2.07	D
S30L-22	−63.33	82.66	−28.22	−32.05	−40.95	0.51	M
C_{60}–C_{60}	−6.33	16.46	−2.02	−14.71	−6.60	2.32	D
C_{60}@[6]CPPA	−45.58	112.74	−13.35	−101.09	−47.29	2.22	D
Cor–C_{60}	−11.64	29.31	−3.38	−29.57	−15.28	2.54	D
Coran–C_{60}	−1.81	4.70	−0.75	−12.91	−10.78	7.13	D
Suman–C_{60}	−19.80	47.62	−6.20	−41.72	−20.10	2.11	D
3NSuman–C_{60}	−16.79	42.63	−4.83	−38.06	−17.04	2.27	D
PClCoran–C_{60}	−16.89	43.72	−3.92	−43.63	−20.72	2.58	D
DClCoran–C_{60}	−18.68	48.07	−4.51	−48.66	−23.78	2.60	D

TABLE S6: Binding energies (kcal/mol) for the vL27 dataset, along with the corresponding mean absolute errors (MAE), root-mean-square errors (RMSE), mean signed errors (MSE), and maximum absolute errors (MAX), computed using MP2-based methods. All MP2 calculations, except for MP2+D3-ML and κ-MP2, were extrapolated to the CBS limit. MP2+D3-ML and κ-MP2 were performed with the aug-cc-pVTZ basis set. BSSE corrections were applied to all MP2 results using the counterpoise method.

vL27	MP2/CBS	SCS-MP2	SCS(MI)-MP2	MP2D	SCS-MP2D	κ-MP2	MP2+D3-ML
CiM-a	−42.20	−35.95	−40.20	−40.97	−41.28	−40.31	−39.69
CiM-c	−19.73	−14.25	−15.71	−16.26	−16.10	−14.23	−15.22
CiM-d	−76.21	−63.58	−71.44	−74.92	−75.33	−71.56	−71.47
CiM-e	−74.72	−55.81	−64.83	−67.80	−68.42	−64.51	−64.49
DNA-ellipticine	−55.60	−37.90	−41.90	−42.15	−41.31	−38.08	−40.67
S12L-3a	−57.30	−39.37	−43.78	−43.85	−42.89	−40.19	−40.76
S12L-4a	−79.85	−54.97	−60.21	−55.38	−52.31	−47.04	−38.73
S12L-5a	−48.60	−38.67	−43.83	−44.70	−45.11	−43.09	−43.91
S12L-5b	−36.32	−27.14	−31.56	−31.76	−31.94	−30.02	−30.01
S12L-7b	−32.78	−22.88	−25.90	−28.48	−28.65	−25.50	−30.71
S30L-5	−60.33	−42.36	−47.67	−44.62	−43.80	−41.64	−38.47
S30L-6	−50.53	−33.55	−38.23	−35.44	−34.75	−32.21	−31.60
S30L-7	−67.61	−44.57	−48.79	−44.74	−42.61	−37.51	−34.13
S30L-8	−76.26	−50.60	−55.34	−50.81	−48.44	−42.49	−39.30
S30L-13	−35.09	−25.33	−28.05	−29.49	−29.47	−26.76	−29.58
S30L-14	−40.11	−28.20	−31.34	−31.08	−31.15	−28.76	−32.04
S30L-19	−18.71	−13.76	−15.48	−18.15	−18.06	−15.51	−18.26
S30L-20	−22.92	−15.63	−17.94	−22.06	−21.89	−18.04	−22.87
S30L-22	−45.84	−39.89	−44.14	−44.49	−44.93	−44.44	−41.97
C_{60}–C_{60}	−20.22	−13.87	−15.18	−14.62	−13.33	−10.79	−7.51
C_{60}@[6]CPPA	−90.92	−59.11	−65.46	−58.11	−54.39	−50.12	−37.57
Cor–C_{60}	−35.11	−24.04	−26.46	−24.45	−22.85	−19.94	−15.15
Coran–C_{60}	−18.99	−14.19	−15.47	−14.87	−14.21	−12.16	−10.73
Suman–C_{60}	−39.86	−26.32	−29.23	−27.23	−25.61	−23.15	−17.12
3NSuman–C_{60}	−34.78	−22.69	−25.24	−23.64	−22.22	−20.32	−15.18
PClCoran–C_{60}	−44.66	−29.83	−32.75	−29.63	−27.81	−25.23	−19.85
DClCoran–C_{60}	−53.03	−35.80	−39.43	−35.23	−33.13	−30.39	−24.72
RMSE	19.79	7.30	8.97	6.53	5.19	3.21	2.24
MAE	15.25	5.94	6.58	4.85	3.94	2.35	1.94
MAX	49.68	17.88	24.23	16.88	13.16	8.89	3.97
MSE	−15.25	−1.62	−5.52	−4.76	−3.91	−1.02	0.55

TABLE S7: Binding energies (kcal/mol) for the vL27 dataset, along with the corresponding mean absolute errors (MAE), root-mean-square errors (RMSE), mean signed errors (MSE), and maximum absolute errors (MAX), computed using selected B97-type functionals combined with the nonlocal VV10 correlation functional. All DFT calculations were performed with the def2-TZVPPD basis set and applied counterpoise corrections.

vL27	ωB97X-V	ωB97M-V	B97M-V	B97M-rV
CiM-a	−40.38	−40.88	−40.70	−41.19
CiM-c	−16.17	−16.53	−15.45	−16.17
CiM-d	−74.75	−76.59	−75.37	−76.77
CiM-e	−71.35	−73.85	−72.09	−74.53
DNA-ellipticine	−42.27	−43.81	−41.34	−43.63
S12L-3a	−41.87	−44.09	−42.06	−44.25
S12L-4a	−39.50	−43.58	−38.48	−40.66
S12L-5a	−43.59	−45.32	−42.60	−43.59
S12L-5b	−30.22	−31.59	−30.39	−31.34
S12L-7b	−34.39	−34.16	−33.20	−35.18
S30L-5	−40.29	−42.91	−40.05	−41.91
S30L-6	−30.26	−32.95	−30.35	−31.92
S30L-7	−35.77	−38.72	−34.96	−37.17
S30L-8	−41.01	−44.05	−39.88	−42.40
S30L-13	−32.77	−33.35	−31.53	−33.23
S30L-14	−34.33	−35.71	−33.41	−35.26
S30L-19	−20.97	−20.71	−20.46	−21.46
S30L-20	−25.74	−25.90	−25.69	−27.06
S30L-22	−44.89	−44.96	−44.20	−44.74
C_{60}–C_{60}	−8.07	−8.92	−8.17	−8.69
C_{60}@[6]CPPA	−38.34	−44.65	−36.19	−38.65
Cor–C_{60}	−16.06	−17.68	−15.59	−16.52
Coran–C_{60}	−12.01	−11.97	−11.28	−11.89
Suman–C_{60}	−18.45	−21.17	−18.35	−19.36
3NSuman–C_{60}	−16.35	−18.64	−16.41	−17.38
PClCoran–C_{60}	−20.48	−23.07	−20.06	−21.29
DClCoran–C_{60}	−24.31	−27.26	−23.43	−24.87
RMSE	2.80	3.63	2.77	3.52
MAE	2.11	2.97	2.25	2.51
MAX	6.66	6.76	5.47	7.45
MSE	−1.04	−2.84	−0.56	−2.02

TABLE S8: Binding energies (kcal/mol) for the vL27 dataset, along with the corresponding mean absolute errors (MAE), root-mean-square errors (RMSE), mean signed errors (MSE), and maximum absolute errors (MAX), computed using selected B97-type functionals with dispersion corrections. All DFT calculations were performed with the def2-TZVPPD basis set and applied counterpoise corrections.

vL27	ωB97X-D	ωB97X-D3	ωB97X-D4	ωB97X-D4rev	ωB97M-D4	ωB97M-D4rev	B97M-D4
CiM-a	−41.24	−41.34	−41.05	−40.62	−40.68	−40.09	−41.35
CiM-c	−16.82	−16.34	−14.62	−14.26	−14.93	−15.10	−14.50
CiM-d	−75.08	−76.39	−70.65	−70.01	−71.71	−70.49	−71.87
CiM-e	−68.58	−69.55	−65.11	−63.77	−66.34	−65.51	−67.13
DNA-ellipticine	−42.57	−40.61	−38.03	−36.62	−38.98	−39.85	−38.88
S12L-3a	−39.77	−38.00	−37.22	−35.45	−37.81	−39.06	−38.81
S12L-4a	−40.34	−33.36	−36.75	−34.13	−37.75	−40.62	−36.96
S12L-5a	−44.41	−43.67	−42.42	−41.59	−42.98	−42.64	−41.71
S12L-5b	−30.08	−30.03	−29.22	−28.45	−29.58	−29.49	−29.70
S12L-7b	−33.66	−32.29	−23.76	−23.09	−24.06	−24.32	−23.67
S30L-5	−40.83	−38.40	−38.25	−36.46	−38.97	−40.73	−39.39
S30L-6	−31.78	−29.71	−29.57	−27.78	−30.05	−31.68	−30.61
S30L-7	−37.88	−35.26	−37.68	−34.99	−36.84	−39.56	−37.68
S30L-8	−43.30	−40.71	−42.82	−39.95	−42.05	−45.10	−42.86
S30L-13	−32.65	−31.81	−25.20	−24.50	−26.00	−26.27	−25.06
S30L-14	−32.69	−33.29	−27.71	−26.43	−27.86	−29.09	−27.55
S30L-19	−20.80	−20.67	−16.04	−15.93	−16.21	−15.74	−16.07
S30L-20	−26.62	−25.68	−18.53	−18.38	−19.30	−18.58	−19.28
S30L-22	−45.61	−45.68	−45.05	−44.66	−44.44	−43.92	−44.43
C_{60}–C_{60}	−7.74	−6.23	−8.29	−7.63	−8.08	−8.68	−8.41
C_{60}@[6]CPPA	−37.89	−30.50	−41.42	−36.78	−39.88	−43.98	−38.45
Cor–C_{60}	−15.68	−13.13	−14.77	−13.65	−15.08	−16.34	−14.84
Coran–C_{60}	−11.32	−11.15	−9.46	−9.32	−9.99	−10.37	−9.56
Suman–C_{60}	−19.00	−14.97	−18.51	−16.73	−18.42	−19.89	−18.33
3NSuman–C_{60}	−16.59	−13.37	−15.80	−14.39	−16.03	−17.07	−16.02
PClCoran–C_{60}	−19.37	−16.94	−19.29	−17.51	−19.18	−21.26	−19.23
DClCoran–C_{60}	−21.92	−19.45	−22.04	−20.15	−22.31	−24.65	−21.83
RMSE	2.65	3.78	1.79	2.99	1.52	1.68	1.75
MAE	2.02	2.97	1.44	2.71	1.28	1.33	1.41
MAX	5.92	10.73	3.98	5.30	3.67	3.67	4.07
MSE	−1.03	0.66	1.38	2.71	1.15	0.24	1.19

TABLE S9: Binding energies (kcal/mol) for the vL27 dataset, along with the corresponding mean absolute errors (MAE), root-mean-square errors (RMSE), mean signed errors (MSE), and maximum absolute errors (MAX), computed using selected Minnesota functionals as well as density functionals combined with D4 and MBD dispersion corrections. All DFT calculations were performed with the def2-TZVPPD basis set and applied counterpoise corrections.

vL27	B3LYP+D4	PBE0+D4	PBE+D4	PBE0+MBD	MN15	M06L-D4	M06-2X-D3	PW6B95-D4
CiM-a	−43.35	−43.07	−42.56	−43.79	−35.60	−41.44	−40.54	−39.65
CiM-c	−15.37	−14.84	−14.46	−15.60	−14.27	−15.67	−14.72	−14.03
CiM-d	−74.54	−72.96	−70.36	−76.94	−68.45	−78.59	−75.34	−70.14
CiM-e	−68.88	−64.81	−61.55	−67.69	−64.03	−74.73	−70.13	−63.11
DNA-ellipticine	−43.43	−37.48	−36.48	−36.49	−37.40	−43.05	−36.44	−35.12
S12L-3a	−42.00	−36.04	−35.23	−34.96	−37.45	−42.69	−38.19	−36.54
S12L-4a	−43.37	−37.27	−34.94	−31.45	−29.91	−37.27	−32.46	−31.19
S12L-5a	−43.12	−42.70	−40.17	−43.98	−40.10	−41.76	−44.51	−40.13
S12L-5b	−31.06	−29.53	−28.32	−30.09	−26.52	−29.43	−29.76	−26.41
S12L-7b	−26.63	−24.99	−23.98	−28.58	−28.26	−36.34	−25.65	−24.08
S30L-5	−43.33	−38.30	−36.88	−35.15	−37.15	−41.16	−38.60	−35.80
S30L-6	−33.93	−30.06	−28.94	−27.70	−27.64	−31.68	−29.19	−25.15
S30L-7	−41.88	−35.52	−35.13	−36.32	−33.60	−36.27	−33.65	−30.77
S30L-8	−47.50	−40.81	−40.49	−41.92	−37.72	−41.30	−37.49	−34.93
S30L-13	−27.30	−25.51	−24.22	−26.97	−31.72	−36.03	−27.90	−24.70
S30L-14	−30.41	−27.41	−25.78	−26.99	−34.34	−38.79	−30.38	−26.36
S30L-19	−16.76	−16.82	−16.68	−19.15	−18.81	−23.32	−17.76	−16.17
S30L-20	−19.77	−19.59	−19.28	−23.69	−23.08	−29.56	−21.92	−19.19
S30L-22	−46.71	−46.85	−46.04	−47.77	−40.56	−45.25	−44.11	−43.79
C_{60}–C_{60}	−9.48	−8.50	−8.42	−6.68	−3.48	−6.64	−5.99	−7.00
C_{60}@[6]CPPA	−44.82	−38.01	−35.63	−33.44	−29.64	−36.15	−34.66	−30.29
Cor–C_{60}	−17.85	−15.52	−14.72	−13.11	−10.69	−13.59	−11.94	−12.04
Coran–C_{60}	−11.67	−11.10	−10.70	−9.88	−7.59	−10.34	−7.53	−8.29
Suman–C_{60}	−20.31	−17.74	−16.76	−15.57	−16.21	−18.11	−17.20	−15.89
3NSuman–C_{60}	−17.66	−15.06	−14.30	−13.41	−13.60	−16.28	−15.10	−12.97
PClCoran–C_{60}	−22.81	−19.13	−18.07	−14.93	−15.63	−18.97	−17.03	−15.88
DClCoran–C_{60}	−26.67	−22.39	−20.94	−17.41	−17.88	−21.89	−18.69	−18.19
RMSE	2.49	1.81	2.94	3.60	4.93	4.55	3.32	4.67
MAE	1.92	1.61	2.57	2.86	4.17	3.57	2.54	4.02
MAX	5.20	3.22	5.60	7.99	11.59	9.83	6.97	10.94
MSE	−1.63	1.28	2.42	1.73	3.15	−1.48	1.84	4.02

TABLE S10: Binding energies (kcal/mol) for the vL27 dataset, along with the corresponding mean absolute errors (MAE), root-mean-square errors (RMSE), mean signed errors (MSE), and maximum absolute errors (MAX), computed using the double-hybrid density functional PWPB95-D4 with def2-TZVPPD, def2-TZVPP, and def2-QZVPP basis sets, as well as at the CBS limit extrapolated from def2-TZVPP and def2-QZVPP. All DFT calculations include counterpoise corrections.

vL27	def2-TZVPPD	def2-TZVPP	def2-QZVPP	CBS
CiM-a	−39.22	−38.91	−39.52	−39.89
CiM-c	−14.48	−14.26	−14.64	−14.76
CiM-d	−69.10	−68.49	−69.53	−70.21
CiM-e	−62.07	−60.83	−62.29	−63.13
DNA-ellipticine	−35.66	−34.53	−35.95	−36.44
S12L-3a	−37.03	−36.22	−37.36	−37.76
S12L-4a	−36.13	−35.51	−36.62	−36.93
S12L-5a	−40.41	−39.96	−40.68	−41.08
S12L-5b	−26.87	−26.44	−27.10	−27.44
S12L-7b	−23.07	−22.49	−23.00	−23.23
S30L-5	−36.70	−35.83	−37.16	−37.63
S30L-6	−26.72	−26.04	−27.20	−27.63
S30L-7	−33.37	−32.38	−34.11	−34.62
S30L-8	−37.74	−36.61	−38.54	−39.10
S30L-13	−24.36	−23.71	−24.50	−24.75
S30L-14	−26.36	−25.55	−26.55	−26.86
S30L-19	−15.61	−15.30	−15.61	−15.81
S30L-20	−18.58	−18.07	−18.59	−18.84
S30L-22	−43.18	−42.97	−43.49	−43.82
C_{60}–C_{60}	−8.74	−8.63	−8.80	−8.86
C_{60}@[6]CPPA	−37.18	−36.42	−37.55	−38.04
Cor–C_{60}	−14.51	−14.22	−14.69	−14.84
Coran–C_{60}	−9.04	−8.84	−9.04	−9.09
Suman–C_{60}	−18.49	−18.17	−18.71	−18.90
3NSuman–C_{60}	−15.55	−15.21	−15.76	−15.93
PClCoran–C_{60}	−18.95	−18.51	−19.25	−19.51
DClCoran–C_{60}	−22.23	−21.75	−22.46	−22.82
RMSE	3.07	3.68	2.80	2.45
MAE	2.78	3.36	2.51	2.17
MAX	5.53	6.66	4.81	4.51
MSE	2.78	3.36	2.51	2.17

TABLE S11: Binding energies (kcal/mol) for the vL27 dataset, along with the corresponding mean absolute errors (MAE), root-mean-square errors (RMSE), mean signed errors (MSE), and maximum absolute errors (MAX), computed using the double-hybrid density functional revDSD-PBEP86-D4 with def2-TZVPPD, def2-TZVPP, and def2-QZVPP basis sets, as well as at the CBS limit extrapolated from def2-TZVPP and def2-QZVPP. All DFT calculations include counterpoise corrections.

vL27	def2-TZVPPD	def2-TZVPP	def2-QZVPP	CBS
CiM-a	−39.59	−39.07	−40.25	−41.05
CiM-c	−13.68	−13.32	−13.74	−14.04
CiM-d	−67.77	−66.63	−68.79	−70.26
CiM-e	−59.32	−57.35	−60.15	−62.06
DNA-ellipticine	−35.14	−33.67	−35.26	−36.32
S12L-3a	−34.85	−33.64	−35.05	−36.10
S12L-4a	−39.85	−39.02	−40.15	−40.94
S12L-5a	−39.92	−39.20	−40.45	−41.36
S12L-5b	−27.70	−27.01	−28.10	−28.87
S12L-7b	−21.54	−20.87	−21.57	−22.08
S30L-5	−36.64	−35.43	−36.90	−38.00
S30L-6	−28.47	−27.45	−28.85	−29.86
S30L-7	−37.20	−35.61	−37.50	−38.75
S30L-8	−42.38	−40.59	−42.66	−44.02
S30L-13	−22.88	−22.17	−22.95	−23.48
S30L-14	−24.65	−23.74	−24.75	−25.44
S30L-19	−14.71	−14.21	−14.79	−15.19
S30L-20	−16.80	−16.16	−16.92	−17.45
S30L-22	−43.25	−42.84	−43.83	−44.54
C_{60}–C_{60}	−10.73	−10.59	−10.81	−10.96
C_{60}@[6]CPPA	−43.26	−42.06	−43.91	−45.15
Cor–C_{60}	−17.04	−16.68	−17.19	−17.54
Coran–C_{60}	−10.92	−10.71	−10.88	−11.01
Suman–C_{60}	−19.38	−18.90	−19.65	−20.15
3NSuman–C_{60}	−16.88	−16.41	−17.10	−17.55
PClCoran–C_{60}	−21.14	−20.48	−21.36	−21.98
DClCoran–C_{60}	−24.88	−24.05	−25.12	−25.87
RMSE	3.15	3.88	2.92	2.45
MAE	2.36	3.03	2.18	1.90
MAX	7.78	9.76	6.96	5.66
MSE	2.07	2.91	1.77	0.98

TABLE S12: Binding energies (kcal/mol) for the vL27 dataset, along with the corresponding mean absolute errors (MAE), root-mean-square errors (RMSE), mean signed errors (MSE), and maximum absolute errors (MAX), computed using the double-hybrid density functional ωB97M(2) with def2-TZVPPD, def2-TZVPP, and def2-QZVPP basis sets, as well as at the CBS limit extrapolated from def2-TZVPP and def2-QZVPP. All DFT calculations include counterpoise corrections.

vL27	def2-TZVPPD	def2-TZVPP	def2-QZVPP	CBS
CiM-a	−40.03	−39.03	−40.50	−41.25
CiM-c	−15.22	−14.83	−12.14	−12.42
CiM-d	−72.43	−71.07	−71.76	−73.09
CiM-e	−67.37	−64.67	−65.93	−67.67
DNA-ellipticine	−40.13	−38.67	−39.37	−40.39
S12L-3a	−41.64	−40.24	−41.28	−42.28
S12L-4a	−46.56	−45.30	−46.87	−47.58
S12L-5a	−43.29	−41.93	−42.43	−43.23
S12L-5b	−30.53	−29.20	−29.88	−30.57
S12L-7b	−29.93	−29.05	−29.74	−30.24
S30L-5	−41.17	−39.82	−40.30	−41.28
S30L-6	−31.91	−30.84	−31.92	−32.78
S30L-7	−37.69	−36.09	−36.89	−37.99
S30L-8	−41.96	−41.17	−41.81	−43.01
S30L-13	−29.08	−28.17	−29.09	−29.59
S30L-14	−31.36	−30.35	−30.21	−30.84
S30L-19	−18.27	−17.55	−18.22	−18.64
S30L-20	−22.05	−21.40	−22.02	−22.56
S30L-22	−43.79	−43.35	−43.21	−43.82
C_{60}–C_{60}	−10.99	−10.84	−11.05	−11.16
C_{60}@[6]CPPA	−48.27	−47.12	−48.94	−50.00
Cor–C_{60}	−19.15	−18.79	−20.35	−20.68
Coran–C_{60}	−12.25	−12.04	−12.78	−12.88
Suman–C_{60}	−22.39	−21.93	−22.53	−22.93
3NSuman–C_{60}	−19.83	−19.38	−19.76	−20.14
PClCoran–C_{60}	−24.63	−24.00	−25.55	−26.10
DClCoran–C_{60}	−29.78	−28.98	−30.76	−31.43
RMSE	2.79	2.42	3.06	3.42
MAE	2.14	1.91	2.37	2.56
MAX	7.13	5.89	7.71	8.77
MSE	−1.68	−0.72	−1.44	−2.15

TABLE S13: Binding energies (kcal/mol) for the vL27 dataset, along with the corresponding mean absolute errors (MAE), root-mean-square errors (RMSE), mean signed errors (MSE), and maximum absolute errors (MAX), computed using five composite electronic-structure methods.

vL27	HF-3c	PBEh-3c	B97-3c	r^2SCAN-3c	ωB97X-3c
CiM-a	−43.69	−37.95	−40.77	−40.18	−41.01
CiM-c	−14.76	−14.29	−14.86	−15.15	−14.70
CiM-d	−69.36	−65.64	−66.71	−70.06	−70.43
CiM-e	−66.30	−56.38	−59.13	−65.52	−63.40
DNA-ellipticine	−40.42	−35.64	−37.80	−39.48	−37.68
S12L-3a	−37.03	−36.42	−37.77	−40.51	−37.94
S12L-4a	−38.34	−36.59	−36.07	−33.38	−35.70
S12L-5a	−39.08	−39.30	−40.25	−42.50	−41.81
S12L-5b	−27.70	−24.59	−27.89	−29.20	−28.83
S12L-7b	−27.79	−22.09	−25.53	−27.25	−26.25
S30L-5	−41.87	−38.30	−39.51	−39.09	−39.26
S30L-6	−32.70	−28.69	−32.00	−29.97	−29.44
S30L-7	−41.12	−32.83	−36.60	−35.06	−36.17
S30L-8	−46.63	−36.86	−41.90	−40.09	−41.59
S30L-13	−29.05	−25.22	−26.79	−28.07	−26.56
S30L-14	−30.68	−27.12	−28.82	−28.75	−27.20
S30L-19	−18.16	−18.30	−16.82	−17.88	−15.99
S30L-20	−22.63	−21.15	−19.47	−20.94	−17.73
S30L-22	−47.10	−44.68	−44.85	−43.80	−45.39
C_{60}–C_{60}	−7.67	−7.30	−7.60	−7.60	−8.10
C_{60}@[6]CPPA	−42.10	−38.61	−36.78	−33.80	−37.77
Cor–C_{60}	−15.52	−14.36	−15.37	−13.85	−14.96
Coran–C_{60}	−10.24	−8.84	−10.36	−9.87	−10.75
Suman–C_{60}	−19.34	−18.81	−17.68	−16.60	−16.78
3NSuman–C_{60}	−17.23	−15.81	−15.01	−14.76	−14.57
PClCoran–C_{60}	−20.31	−19.95	−19.70	−17.36	−17.69
DClCoran–C_{60}	−23.18	−22.36	−23.75	−20.14	−20.03
RMSE	2.20	3.92	2.79	2.79	2.31
MAE	1.81	2.98	2.05	2.10	1.94
MAX	4.80	10.72	7.98	7.44	4.98
MSE	−0.13	2.90	1.73	1.69	1.80

TABLE S14: Binding energies (kcal/mol) for the vL27 dataset, along with the corresponding mean absolute errors (MAE), root-mean-square errors (RMSE), mean signed errors (MSE), and maximum absolute errors (MAX), computed selected semi-emprical methods as well as the IPML and CLIFF machine learning potentials.

vL27	g-xTB	GFN2-xTB	DFTB3-D3H5	PM6+D3H4	PM6-ML	IPML	CLIFF
CiM-a	−42.06	−41.90	−49.09	−49.31	−43.03	−46.84	−58.26
CiM-c	−13.00	−13.00	−13.96	−13.26	−13.02	−17.79	−14.54
CiM-d	−71.38	−64.55	−86.89	−79.88	−72.99	−73.87	−83.01
CiM-e	−61.22	−69.01	−78.07	−76.91	−67.88	−77.32	−77.26
DNA-ellipticine	−33.73	−40.02	−38.52	−38.85	−28.16	–	–
S12L-3a	−37.17	−38.40	−39.15	−36.64	−29.16	−43.44	−48.55
S12L-4a	−35.97	−37.51	−34.70	−30.21	−50.51	−47.72	–
S12L-5a	−41.16	−35.47	−40.82	−41.65	−42.81	−46.90	−61.39
S12L-5b	−29.23	−28.05	−30.82	−31.56	−30.53	−37.62	−44.62
S12L-7b	−20.16	−21.95	−34.08	−33.18	−30.14	−38.47	−40.02
S30L-5	−38.76	−39.65	−38.77	−37.43	−35.98	−49.56	−43.43
S30L-6	−31.82	−30.48	−27.52	−26.11	−19.83	−36.05	−29.53
S30L-7	−39.82	−42.62	−37.89	−32.30	−33.90	−41.47	−28.80
S30L-8	−46.23	−49.03	−43.32	−37.05	−36.61	−48.19	−34.45
S30L-13	−22.26	−22.86	−29.79	−30.41	−25.94	−32.60	–
S30L-14	−24.49	−26.20	−33.10	−30.54	−29.41	–	–
S30L-19	−15.59	−14.99	−20.48	−20.52	−16.61	−22.49	−24.42
S30L-20	−19.19	−17.41	−25.88	−25.22	−20.02	−29.38	−30.90
S30L-22	−43.40	−41.71	−45.09	−45.39	−44.77	−45.32	−44.85
C_{60}–C_{60}	−9.44	−10.90	−7.67	−6.57	−17.25	−10.36	–
C_{60}@[6]CPPA	−40.74	−46.49	−37.19	−29.29	−55.87	−46.77	–
Cor–C_{60}	−15.06	−16.37	−14.24	−12.60	−22.78	−19.37	–
Coran–C_{60}	−9.17	−9.14	−9.82	−8.94	−8.69	−11.00	–
Suman–C_{60}	−18.64	−19.24	−16.94	−13.84	−32.74	−22.43	–
3NSuman–C_{60}	−16.98	−17.38	−15.92	−12.97	−32.90	−19.52	–
PClCoran–C_{60}	−20.05	−22.34	−24.67	−17.21	−31.47	–	–
DClCoran–C_{60}	−23.55	−25.82	−27.85	−19.37	−33.58	–	–
RMSE	2.98	3.63	4.42	5.13	7.25	5.95	10.32
MAE	2.36	2.73	3.16	4.24	5.40	4.99	8.81
MAX	7.58	9.61	12.73	11.94	15.46	11.43	18.29
MSE	1.71	0.89	−1.33	1.08	−1.49	−4.88	−6.31
